# No Effects of Predictability on Word-Meaning Priming and Incidental Memory

**DOI:** 10.5334/joc.495

**Published:** 2026-03-31

**Authors:** Vanessa G. Keller, Matthew H. C. Mak, Scott A. Cairney, M. Gareth Gaskell

**Affiliations:** 1 Department of Psychology, University of York, UK; 2 Oxford Centre for Human Brain Activity, Department of Psychiatry, University of Oxford, UK; 3 Department of Psychology, University of Warwick, UK

**Keywords:** Prediction, Memory, Priming, Language Comprehension, Plasticity

## Abstract

Encountering a homonym in a sentential context that biases interpretation towards its subordinate meaning makes that meaning easier to access later. This word-meaning priming effect is not restricted to homonyms and may be supported by general episodic memory processes. Such an account predicts that word-meaning priming may be affected by factors that affect episodic learning (e.g., predictability). Specifically, the contextual predictability of an incoming stimulus has been shown to affect episodic memory in that both highly expected as well as highly unexpected input leads to better memory for that input, via two different underlying neurocognitive mechanisms. We hypothesised that if word-meaning priming is supported by episodic memory processes, both highly expected as well as highly unexpected target words should lead to stronger word-meaning priming effects than target words of intermediate predictability. Four pre-registered online experiments tested whether contextual predictability affects word-meaning priming and incidental memory for language. We exposed participants to sentences that emphasised a particular aspect of a sentence-final target word’s meaning. Importantly, target words differed in how predictable they were based on the sentence context (e.g., “You can get in a good workout by riding / lifting a bicycle”). Associate production and semantic relatedness judgement assessed the strength of word-meaning priming. Contrary to our hypotheses, while there was evidence for priming across experiments, we found no significant effects of contextual predictability in any experiments. Our findings suggest that predictability-guided updating is rarely triggered during language comprehension, and we outline the theoretical implications of these results for the architecture of the language system.

## 1. Introduction

Over the past two decades, multiple lines of psycholinguistic investigation have provided evidence for an important role of learning mechanisms in support of language comprehension. The reach of adaptive learning processes covers the perception of phonemes (Norris, McQueen, & Cutler, 2003), the articulation of speech sounds according to the linguistic statistics of our environment (Dell, Reed, Adams, & Meyer, 2000), the acquisition and consolidation of novel word forms and meanings (Gaskell & Dumay, 2003; Rodd et al., 2012), as well as adaptation to new syntactic structures (Ball, Mak, Ryskin, Curtis, Rodd, & Gaskell, 2025; Kaschak & Glenberg, 2004; Ryskin, Qi, Duff, & Brown-Schmidt, 2017). At the same time, there has been a proliferation of psycholinguistic research that has applied specific theories from the wider memory literature to the study of human language processing and comprehension (e.g., Davis & Gaskell, 2009; Gagnepain, Henson, & Davis, 2012). One particular example comes from an area of psycholinguistics concerned with lexical-semantic processing, where different theories of memory have been applied and experimentally tested in an attempt to accurately describe the underlying mechanism supporting a highly flexible and widespread type of adaptation or ‘re-tuning’ of the mental lexicon.

Specifically, previous research has shown that encountering a lexically ambiguous word in a sentential context that biases its interpretation towards a subordinate meaning leads to that meaning subsequently being more easily accessible (e.g., Rodd et al., 2013, 2016; Gilbert et al., 2021). This finding, referred to as *word-meaning priming*, illustrates the malleability of stored word meanings. The word-meaning priming effect can be detected minutes or even hours later, with its longevity clearly distinguishing it from shorter-term semantic priming (e.g., Rodd et al., 2013, 2016). In a standard word-meaning priming study, participants hear or read sentences containing lexically ambiguous words in contexts that bias interpretation towards the subordinate meaning (e.g., “The most recent opinion polls did not favour the current *party*.” – interpretation: a group of people organized to acquire and exercise political power). When participants are subsequently asked to generate semantic associates in response to isolated ambiguous words (e.g., ‘party’), the proportion of context-consistent answers (e.g., ‘politics’) increases for homonyms that were previously encountered with their subordinate meaning compared to unprimed ambiguous words.

Recent years have seen a substantial amount of work investigating the time course and constraints on this type of lexical-semantic updating in order to shed light on the underlying mechanism (e.g., Gaskell, Cairney, & Rodd, 2019; Gilbert et al., 2021; Rodd et al., 2013, 2016). For instance, to evaluate the longevity of the word-meaning priming effect, Rodd et al. (2016) varied and compared latencies between exposure and test (consisting of 1 min, 20 min, and 40 min delays). They found evidence for word-meaning priming at all intervals, but the effect was strongest when the delay was only 1 min. This was taken to suggest that there is likely a rather short-lived component involved in this lexical-semantic updating process, but the presence of word-meaning priming effects after 20 and 40 mins pointed to somewhat more long-lasting alterations to the semantic network. Follow-up experiments mapped out the exact time course of the effect and showed a gradual decay over the course of a day. Beyond that point, the priming effects were weakened and, in some cases, no longer statistically significant (Rodd et al., 2016).

To explain this pattern of results, Rodd et al. (2016) suggested that these longer-term priming effects are best interpreted within a connectionist framework where word forms and ambiguous word meanings are represented in a distributed lexical network (e.g., Rodd, Gaskell, & Marslen-Wilson, 2004). This connectionist network is assumed to model lexical-semantic representations of words in semantic memory and so adjustments to weights as a consequence of language exposure would represent long-term changes in the neocortical language network (and are used to explain neocortical learning more generally in other domains; see e.g., Elman, 2005). By this connectionist account, recent experience with an ambiguous word would lead to direct alteration of the connection weights that map between lexical form and meaning units, strengthening the connection between a word form and the comprehended meaning, which would facilitate the activation of the primed meaning compared to the unprimed one (Rodd et al., 2016). While this model could explain why participants are more likely to retrieve a primed meaning that they have recently been exposed to, it is less clear how it could explain the observed decay function whereby priming becomes weaker during the course of the day, flattening off rather strongly after the first few minutes and then decaying more gradually (Rodd et al., 2016). One possibility would be to ascribe the observed decay to further updating of the connections between other units due to exposure to unrelated language input after the priming event, which might affect the previously strengthened (primed) connection weights. However, it is difficult to explain why decay is stronger at the start and then weakens as time goes by (until priming effects become undetectable).

An alternative way to account for this pattern of decay would be to hypothesise that the lexical-semantic re-tuning observed in the above studies is not the result of direct neocortical changes as would be assumed under a distributed connectionist framework, but of a more flexible, episodic learning system that encodes the association between the primed word and the context in which it was comprehended (see Gaskell, 2024, for further discussion). This would result in the creation of a temporary memory trace that binds together the words in the exposure sentences, which could be used to bias participants’ responses during the test phase towards the meaning in which a prime word was previously interpreted (Gaskell et al., 2019). While the episodic memory system is known to support the rapid encoding of novel associations (as would be required to observe learning effects in a word-meaning priming experiment), these newly formed memory traces have been shown to decay rapidly (Hardt, Nader, & Nadel, 2013). Therefore, the episodic memory system, primarily supported by the hippocampus (Moscovitch, Cabeza, Winocur, & Nadel, 2016; Tulving, & Markowitsch, 1998), might underpin both the priming effects as well as the observed decay over the course of a day that was found by Rodd and colleagues (2016). This alternative explanation is termed the *episodic context* account (Curtis, Mak, Chen, Rodd, & Gaskell, 2022; Mak, Curtis, Rodd, & Gaskell, 2023), given that it hypothesises a crucial role for episodic memory in supporting the updating of word meanings.

To summarise, there are two competing explanations regarding the neurocognitive underpinnings of the word-meaning priming effect. On the one hand, the effect could be the result of direct changes within the existing lexical neocortical network that serve to strengthen the connections between the word form and its comprehended, contextually appropriate meaning (e.g., Gilbert et al., 2018; *immediate alteration* account). On the other hand, the observed lexical-semantic retuning could be driven by the episodic memory system, which binds the ambiguous word to the context it was encountered in, thereby creating a new and perhaps temporary memory trace that may bias participants’ responses towards that specific context at test (Gaskell et al., 2019; *episodic context* account). What differentiates these two accounts is the predictions they make with regard to the influence of a longer consolidation period that includes a sleep interval. If word-meaning priming is supported by the episodic memory system, as predicted by the *episodic context* account, then one would expect sleep-associated memory consolidation processes (involving the replay of memory traces that were acquired during wake; Rasch & Born, 2013; Brodt et al., 2023) to help stabilise the recently acquired memory combining the ambiguous word and the context it was encountered in. Moreover, sleep-associated consolidation would be expected to subtly adjust the stored lexical-semantic representations by reactivating the word form and comprehended meaning during sleep, thereby strengthening the connection between these two units. In contrast, the *immediate alteration* account would not predict any role for sleep-associated consolidation processes considering that all the updating has already taken place immediately after the ambiguous word was encountered in context.

To address this possibility, Gaskell et al. (2019) looked at whether the word-meaning priming effect is better preserved over a period of time involving sleep (potentially enabling episodic/hippocampal memory consolidation) vs. wake. The results indicated that the contextual priming of word meanings is maintained over a consolidation period involving sleep (see also Mak et al., 2023). While these behavioural experiments did not set out to specify the neural substrates underpinning the observed effect, following the reasoning laid out above, the results hint at the possibility that the hippocampus may have been involved, as one would not expect there to be consolidation effects if lexical-semantic updating were supported by immediate alterations of neocortical connections. Instead, in light of their results, Gaskell et al. (2019) proposed that the same hippocampal circuitry responsible for episodic declarative memory tasks (e.g., paired-associate learning) that are typically sensitive to sleep-associated consolidation might also support the updating of word meanings by binding the familiar word form to its context at encoding. Episodic memory is traditionally defined as a type of declarative memory that includes information about the specific context it was encoded in (Tulving, 2002), and the contextually rich memory traces that are assumed to be acquired during comprehension can then be drawn upon in later test phases. This would explain the immediate word-meaning priming effect in that the recently acquired episodic memory biases participants to provide an associate response that is related to the word meaning they have comprehended in earlier sentences (Gaskell et al., 2019). Crucially, during consolidation, this representation may be integrated into existing lexical knowledge structures to optimise future comprehension.

The *episodic context* account thus suggests that word-meaning priming is supported by general episodic memory processes, whereby word meanings are initially bound to the context they were encountered in to form an episodic memory trace. By this account, the encoded episodic representation of the linguistic input serves as an additional source of information to guide later interpretation of a previously comprehended word, and may further be used to update stored language knowledge via sleep-associated consolidation. This serves to ensure that the stored representations accurately reflect the statistics of the linguistic environment and allows listeners to optimise future interpretations.

One prediction of the *episodic context* account is that this updating of lexical-semantic information should extend beyond homonyms (words with multiple meanings), considering the mechanisms supporting such updating are assumed to be general episodic memory processes (Gaskell et al., 2019). Indeed, the word-meaning priming effect has recently been extended to non-homonyms (i.e., to words that are not ambiguous in the sense described above, and that have only one dictionary entry), where subsequent interpretation of the word becomes biased towards the previously comprehended contextually appropriate aspect of the word’s meaning (e.g., either the ‘cleanliness’ or ‘relaxation’ aspect relating to the word *bathtub*; Curtis et al., 2022). This suggests that the lexical-semantic updating observed within the word-meaning priming paradigm might be a general learning mechanism that operates whenever words are comprehended in context, which would point to an important role for episodic memory in language comprehension (Curtis et al., 2022). Moreover, lexical-semantic updating in response to encountering non-homonyms in sentential contexts is stronger if exposure and testing is separated by a night of sleep compared to a day awake (Mak et al., 2023), which further points towards a general role of episodic memory with regard to the continual refinement of stored word meanings. Finally, recent evidence shows that comprehending even two unrelated words together in a sentential context alters their later interpretation in that they are subsequently perceived as being more related even 12 hours after initial exposure (Mak, Ball, O’Hagan, Walsh, & Gaskell, 2024). This provides strong evidence for the involvement of episodic memory in language comprehension.

Recent research into the time course and underlying mechanisms of the word-meaning priming effect has thus accumulated some evidence for an important role of episodic memory processes. If this type of lexical-semantic updating is assumed to be supported by episodic memory processes, then perhaps a useful way to further test this hypothesis is by looking to results from other episodic memory paradigms and neurocognitive models of episodic memory, with the aim of testing whether factors known to influence episodic memory in other paradigms might also influence word-meaning priming. This might provide indirect evidence that episodic memory underpins not only the encoding of novel stimuli or associations, but that it also supports the updating of stored word meanings (Gaskell et al., 2019) or meaning aspects (Curtis et al., 2022) by encoding a memory trace of the comprehended meaning that is bound to the context it was experienced in (as predicted by the *episodic context* account; Gaskell et al., 2019).

If word-meaning priming is supported by episodic memory processes, the strength of word-meaning priming effects might be affected by factors that have previously been shown to affect learning in (other) episodic memory paradigms (e.g., Greve et al., 2019; Quent et al., 2022). One important finding in this regard (replicated across several different paradigms, e.g., Greve et al., 2019; Quent et al., 2022; van Kesteren et al., 2013) is that participants tend to be better at remembering highly expected as well as highly unexpected material relative to material of intermediate expectedness. Numerous studies have provided evidence for better memory for both information that fits a pre-established schema and is highly expected (e.g., Craik & Tulving, 1975) as well as information that is incongruent with prior knowledge and is highly unexpected (e.g., von Restorff, 1933). One influential neurocognitive model in the field of episodic memory that aims to account for these results is the SLIMM model (first introduced by van Kesteren, Ruiter, Fernandez, & Henson, 2012), which stands for *schema-linked interactions between medial prefrontal cortex (mPFC) and medial temporal lobes (MTL)*, the latter of which prominently include the hippocampi (see also Henson & Gagnepain, 2010, for a related model and Quent, Henson, & Greve, 2021, for a recent review of the findings supporting these models).

On a neural level, SLIMM proposes that the mPFC and the MTL/hippocampus have different roles to play in encoding episodic memories depending on how expected the event or item to be encoded is (or, in the original formulation, whether it fits with a pre-existing schema, i.e., a structure of prior knowledge that has been built up with experience; van Kesteren et al., 2012). Events or items that are (highly) expected are assumed to benefit from very rapid encoding and direct consolidation into neocortex, which is supported by the mPFC. In contrast, events or items that are highly unexpected are assumed to be reliant on the hippocampus/MTL, which encodes a ‘snapshot’ of the entire context that can later be consolidated via hippocampal memory consolidation (Quent, Greve, & Henson, 2022). This prediction has been (at least partially) confirmed in previous studies using functional magnetic resonance imaging (fMRI; van Kesteren, Fernandez, Norris, & Hermans, 2010; Henson & Gagnepain, 2010). For instance, van Kesteren et al. (2010) found that encoding information for which participants had a strong prior schema (compared to no schema) resulted in greater intersubject synchronisation within the mPFC and less hippocampal-mPFC interregional connectivity. This suggests that the mPFC preferentially encodes information that conforms to a pre-existing schema, whereas the hippocampus steps in to encode schema-incongruent or unexpected information.

SLIMM thus explains the fact that both highly expected as well as highly unexpected input has a memory advantage by postulating that different brain systems underpin these two ends of the expectedness scale. It should be noted, however, that it is likely that both memory systems are involved in the encoding of information across the range of possible expectancy levels, with the activation and support provided by the mPFC and hippocampus for the encoding of expected vs. unexpected information being a matter of degree. That being said, the U-shaped function of memory against the expectancy of the information to be encoded has been well replicated (e.g., Greve, Cooper, Kaula, Anderson, & Henson, 2017; Greve, Cooper, Tibon, & Henson, 2019; Quent et al., 2022). For instance, most recently, Quent et al. (2022) used an immersive virtual reality paradigm to assess how the expectedness of a stimulus influences episodic memory. While virtually moving around in a kitchen, participants were presented with 20 familiar objects that were placed in either highly expected, neutral, or highly unexpected locations (e.g., for a kettle, an expected location would be the kitchen counter, a neutral location the kitchen table, and an unexpected location the sink). In a subsequent test, participants were better able to recall the location of items placed in highly expected as well as highly unexpected places (compared to neutral ones), showing a U-shaped curve of memory performance against expectedness (Quent et al., 2022).

Building on the empirical results from the memory literature outlined above, in the current experiments, we manipulated the expectedness of the target word in a word-meaning priming experiment in an attempt to further elucidate the involvement of episodic memory in lexical-semantic updating. It should be noted at this point that while the role of prediction for the processing of language has been well studied (see e.g., Ryskin & Nieuwland, 2023; Kuperberg & Jaeger, 2016; for reviews), the influence of expected or unexpected linguistic input on memory is less well understood (Ryskin & Niewland, 2023). This has led us to look to well-established models within the episodic memory literature from which to derive our predictions as to the role of episodic memory processes in lexical-semantic updating.

This paper presents three priming (Exp. 1–3) and one non-priming (Exp. 4) experiments. In the former, participants were exposed to sentences containing a target word whose interpretation was biased towards a specific aspect of its meaning by the preceding sentential context (i.e., the word-meaning priming manipulation). These sentence-final target words were either highly expected based on the preceding sentence context (Expected condition), highly unexpected based on the preceding sentence context (Unexpected condition), or of intermediate expectedness, in which case the lead-in sentence did not encourage any specific strong predictions to be made (Neutral context). If word-meaning priming is indeed supported by episodic memory processes, then the strength of the word-meaning priming effect might be affected by a factor that has previously been shown to affect learning in other episodic memory paradigms, i.e., the expectedness of the stimulus (e.g., Greve et al., 2019; Quent et al., 2022). We thus predicted a greater word-meaning priming effect in the case where participants encounter the prime word in the Expected and Unexpected condition, compared to the Neutral condition which does not allow for strong lexical predictions to be made. Therefore, we expected to see a U-shaped curve of priming against the expectedness of the prime word. Additionally, while we predicted that both highly expected as well as highly unexpected items should lead to stronger priming than ‘neutral’ items, we anticipated that the effect would be strongest for highly unexpected items since we assumed that the hippocampus should be crucially involved in the updating of stored word meanings.

A subsidiary aim of the current paper was to test two further predictions derived from the episodic memory models outlined above. The first was that memory for incidental aspects of the materials would be better if these features were processed in close temporal proximity to unexpected input (compared to expected or neutral input). In all three priming experiments as well as in the non-priming experiment, we used a combination of cued recall and two-alternative-forced-choice (2AFC) tests probing memory for incidental aspects of the experiments. The second prediction was that memory for highly expected as well as highly unexpected items would be better than memory for neutral items. We tested this prediction in the final (non-priming) experiment using an old/new recognition memory test assessing memory for the sentence-final words.

## 2. Experiment 1

This experiment, as well as all the following ones, was pre-registered prior to data collection. The pre-registration (https://osf.io/x4nfu) contains the exclusion criteria and analysis plan, and deviations from this plan (including additional exploratory analyses) are explicitly mentioned throughout. The data and analysis scripts for all the experiments presented here are publicly available on the Open Science Framework (https://osf.io/a8mj4/?view_only=0fe94de6d5ce4000975ca304945a6b91).

### 2.1 Methods

#### 2.1.1 Participants

Ninety-nine participants, aged between 18 and 35 years, were recruited online via Prolific. They were paid at the rate of £7 per hour. They were all native speakers of English and reported not to have any language-related or attentional disorders. Our pre-registered target sample size was 80 usable datasets, based on the recommendation by Brysbaert and Stevens (2018) to aim for 1600 observations per condition when using (generalised) linear mixed-effects models for statistical analysis (80 participants × 20 targets per condition = 1600 observations per condition). Following our pre-registered exclusion criteria, we discarded the data from 19 participants in total (six due their performance in the filler task, six due to low accuracy in the relatedness judgement task, and 7 due to misunderstanding or failing the attention checks in the associate production task), resulting in 80 usable datasets (42 female; mean age = 29.0, *SD* = 4.72).

#### 2.1.2 Materials

##### 2.1.2.1 Stimuli

The first experiment contained 80 target words in total, with three sentences per target. For the entire set of stimuli, the three sentences had the same final target word but differed in expectedness: Expected (i.e., the preceding sentence context was strongly constraining for one particular final word), Neutral (i.e., the context did not allow for a strong prediction for any particular word), Unexpected (i.e., the context strongly constrained for a specific word; however, instead of that word completing the sentence, a different word was used). The Expected sentences were partially taken and adapted from Curtis et al. (2022). An example sentence from each condition is shown in Table 1.

**Table 1:** Example sentences for the target word ‘concert’ in each of the expectedness conditions, with the mean cloze (0–1) and surprisal (1–10) scores for the selected set of experimental stimuli.


*CONDITION*	*SENTENCE*	*CLOZE SCORE*	*SURPRISAL SCORE*

Expected	While Hannah enjoyed the symphony orchestra’s performance, she was concerned about the fact that her ears were still ringing from last night’s concert.	0.672 (*SD* = 0.261)	1.791 (*SD* = 0.78)

Neutral	To protect her hearing, Hannah decided to buy a pair of high-quality earplugs to wear at the annual concert.	0.125 (*SD* = 0.161)	3.3 (*SD* = 1.49)

Unexpected	Each Bonfire Night, Hannah is concerned about the ear-piercing bangs caused by the spectacular concert.	0.007 (*SD* = 0.029)	6.382 (*SD* = 1.9)


Note that while the conditions differ in how strongly they bias towards a specific word form, all three conditions were intended to be equal in terms of how they biased the interpretation of the sentence-final word’s meaning towards one specific aspect of meaning. In the examples shown in Table 1, all three sentences bias towards the ‘loudness’ interpretation of *concert*, and this is the primary meaning aspect that will be comprehended in all conditions. It is only in the Expected and Unexpected condition, however, that the sentences constrain for a specific word form. It is therefore important to separate the interpretation bias (which should be the same in all three conditions) from the word form identity bias (which is the crucial experimental manipulation and differs between the conditions).

The targets were pseudo-randomly allocated to four different counterbalanced lists, with each target appearing in one of the three expectedness conditions (60 targets in total, 20 per condition, presented during exposure) or the unprimed condition (20, not presented during exposure) in each list. This means that a participant only encountered each target in one of the three expectedness conditions or in the unprimed condition. It should be noted that while the unexpected items were meant to be unpredictable and elicit surprise when encountering the final (target) word, the endings were also designed to be conceivable and not to make the sentence semantically incongruent. Finally, each sentence was followed by two probe words (e.g., *noise* – *pavement*, for the example above), where participants chose which word was more closely related to the sentence they just read. This was meant to ensure that the sentences were interpreted correctly and served as an attention and comprehension check.

##### 2.1.2.2 Norming experiment

The final set of stimuli was derived based on a norming experiment using two independent samples of participants recruited from Prolific (*n* = 46 for cloze ratings, and *n* = 45 for surprisal ratings). Ninety sentence triplets entered the norming experiment, with the goal of ending up with 80 suitable sentence triplets for the main experiment in line with our power calculation (i.e., allowing us to discard the 10 worst-performing items while adhering to our estimated requirement for statistical power). The norming experiment consisted of two separate tasks, a cloze task and a surprisal rating task, both of which are explained below.

One sample of participants (*n* = 46^1^) completed a cloze task where they read a set of sentences that were all missing the final word and were asked to type in the word that they thought best completed the sentence. The sentences were pseudo-randomly allocated to three different lists so that each participant was only exposed to one condition of each stimulus triplet (i.e., to either the expected, neutral, or unexpected sentence ending). On the basis of participants’ responses, a cloze score (see Table 1) was calculated for each sentence indicating how many participants provided the intended target word, divided by the number of responses per item (15 or 16). In the surprisal rating task, a different set of participants (*n* = 45) read a set of sentences where the final word was presented after a delay (the stimuli were again pseudo-randomly allocated to three different lists, as described above). Participants were asked to indicate on a scale of 1–10 how surprised they were to encounter the sentence-final word (i.e., how unexpected the final word was). A surprisal score (see Table 1) was calculated for each sentence as the mean surprisal rating provided by participants (*n* = 15 per individual sentence). There was a statistically significant difference in the mean surprisal ratings between all three conditions (*p*s < .001).

##### 2.1.2.3 Selection of the final stimuli

The final set of stimuli was selected by comparing the cloze and surprisal scores of the three sentences in each stimulus triplet (i.e., with an expected, neutral, or unexpected ending), and, if necessary, revising one of the sentences to make it more or less predictive of the final word. Stimulus triplets were included in the final stimulus set if the cloze score for the expected target was higher than for the neutral and unexpected targets. The surprisal scores also needed to differ substantially within each sentence triplet (with the unexpected ending having the highest score, followed by the neutral and then the expected endings). If one of these conditions was not met, at least one of the sentences in a triplet was slightly revised so as to be more or less predictive (according to the authors’ judgement). For instance, the original sentence “Whenever I set the table for dinner, I need to be careful not to let the hand-crafted salad bowls fall out onto the floor when I open the door of the *cupboard*.” was slightly adapted to “Whenever I set the table for dinner, I need to be careful not to let the hand-crafted salad bowls fall out onto the floor when I open the door of the wooden *cupboard*.” in order to be more predictive of the final word. In total, 10.37% of all norming sentences underwent such minor revisions. Finally, the 10 sentence triplets that differed the least in cloze and surprisal scores (or if the difference went in an unexpected direction) were discarded, and the remaining 80 sentence triplets were retained for the main priming experiment.

#### 2.1.3 Design

Experiment 1 consisted of a within-subjects design with one independent variable: Expectedness, which has four levels (Expected, Neutral, Unexpected, and Unprimed). In the unprimed condition, the target word was only shown at test but not presented in the exposure phase. As stated above, each participant encountered each target in only one of the four conditions.

To assess word-meaning priming, participants completed two different tasks: speeded semantic relatedness judgements and associate production. For relatedness judgements, there were two dependent variables: Accuracy (coded as a binary variable, 1 – correct vs. 0 – incorrect) and Response time (in ms). For associate production, there was one dependent variable: Consistency, i.e., how consistent the response produced by participants is with the primed meaning aspect. We explain how Consistency was indexed in detail in the Procedure and Analysis sections. To assess incidental memory, there was one dependent variable: Accuracy (coded as a binary variable, 1 – correct vs. 0 – incorrect).

#### 2.1.4 Procedure

**Figure 1:**
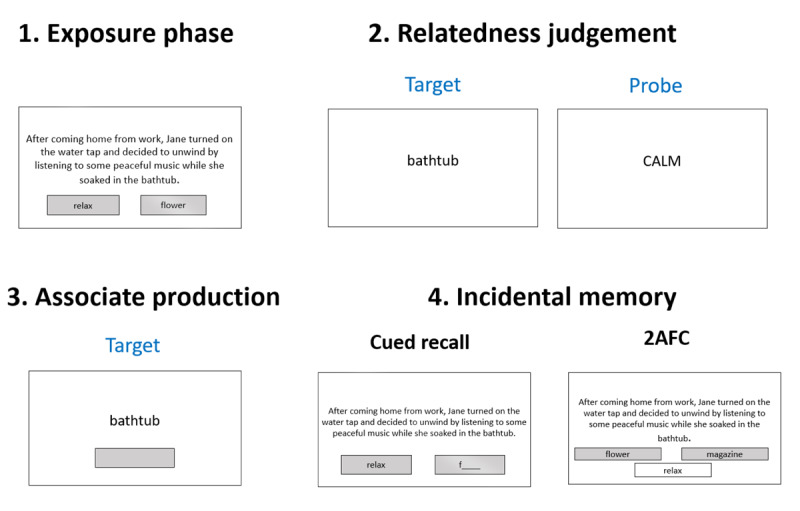
Schematic of the procedure for Experiment 1. Exposure phase and relatedness judgement were separated by a 10-minute clip of “Shaun the Sheep”.

In the three main priming experiments, participants were presented with sentences containing a target word whose interpretation was biased towards a specific aspect of its meaning by the preceding sentential context (i.e., the word-meaning priming manipulation), with the central question being whether target words in the Expected as well as the Unexpected conditions elicit stronger word-meaning priming effects than target words in the Neutral condition. The procedure is detailed in Figure 1, and each part is described below.

##### 2.1.4.1 Exposure phase

During the exposure phase, participants were presented with 60 sentences, each containing a sentence-final target word following a sentential context that biased the word’s interpretation towards a specific aspect of its meaning. Twenty of these target words belonged to the Expected condition, 20 in the Neutral condition, and 20 appeared in the Unexpected condition. Each sentence appeared in its entirety and remained on the screen until participants clicked on the ‘Next’ button, which appeared after 5 seconds on each trial. To ensure that participants were comprehending the sentences with their intended meaning, each sentence was followed by two simultaneously presented probe words, where participants had to choose the one that was related to the sentence they had just read by clicking on it.

##### 2.1.4.2 Filler task

As a filler task, participants watched a 10 min video with minimal linguistic content (an episode of “Shaun the Sheep”; https://www.shaunthesheep.com/), which was followed by three comprehension questions that served as an attention check and assessed whether participants were following the main plot of the Shaun the Sheep episode.

##### 2.1.4.3 Speeded semantic relatedness judgments

In relatedness judgement, there were 120 trials, each featuring a word appearing in isolation for 400 ms (following a 500 ms fixation cross). This was followed by a 200 ms blank screen, after which a probe word appeared (e.g., concert – LOUD). Participants were asked to decide as quickly as possible as to whether the two words are semantically related by pressing a button on their keyboard (F = Related, J = Unrelated), and they were given a maximum of 1500 ms to respond. The words that served as probes in the relatedness judgement task were taken from a previously published word-meaning priming study, which had shown robust priming effects (Curtis et al., 2022). The critical words all corresponded to the primed meaning aspect of the target words. There were 80 trials with target words that were paired with a related probe word and 40 filler trials consisting of words that were not presented during the exposure phase and which were paired with an unrelated probe word. At the start of the task, participants completed six practice trials (3 of which were related, 3 unrelated), where they received immediate feedback on whether their response was correct. No feedback was given in the experimental trials, other than a message reading “Too Slow!” (which appeared for 1500 ms) on trials where participants failed to respond within the maximum time available to them.

##### 2.1.4.4 Associate production

Participants were presented with the 80 target words (each following a 500 ms fixation cross) and were asked to type in the first word that came to mind upon seeing the target word. After giving a response, they pressed the “Return” key on their keyboard to go to the next trial, and it was impossible to move on without providing an answer. Besides the 80 experimental trials, there were also six attention checks which prompted participants to recall and type in the target word from the previous trial. These attention checks were randomly distributed across the associate production task.

##### 2.1.4.5 Incidental memory tests

To assess participants’ memory for incidental aspects of the experiment, and how this may be affected by the relative expectedness of the stimuli, participants were prompted to recall the unrelated word in the exposure phase that followed each sentence. They were presented with the 60 sentences (in their entirety) they encountered during the exposure phase as well as the related word that followed the sentence (presented underneath), and they were asked to type in the unrelated word (submitting their responses by pressing the “Return” key). In a second memory test (2AFC), they were again presented with the exposure sentence and the related word (appearing underneath the sentence), together with the unrelated word and a foil (which also appeared underneath the sentence). They had to choose the unrelated word if they remembered it from the exposure phase by clicking on it. Participants were forced to make a decision to advance to the next trial, meaning they were required to guess the correct answer if they did not remember the word from the exposure phase.

#### 2.1.5 Analysis

For relatedness judgements, there were two dependent variables: Accuracy (coded as a binary variable, 1 – correct vs. 0 – incorrect) and Response time (in ms). For the associate production task, there was one measured variable: Consistency, i.e., how consistent the response produced by participants is with the primed meaning aspect. In Experiment 1, this measure was a continuous variable from –1 to 1 derived from word2vec (Mikolov et al., 2013; Mandera et al., 2017), and indicated the semantic similarity between the response provided by participants and the relatedness task probe word (which was the primed meaning aspect), with more positive values indicating more semantically similar word pairs. As an additional exploratory analysis (not pre-registered), we ran a consistency rating experiment to assess whether the response provided by participants in the associate production task was consistent with the primed meaning aspect. An independent set of third-party raters (*n* = 124 in total; ~24 raters for each target word) were asked to rate whether the response word provided by participants is consistent with the related probe word that was shown in the exposure phase (i.e., the primed meaning aspect). They were asked to give their answer on a scale of 1–10, with 10 indicating that the two words were very closely related or entirely consistent, and 1 indicating that the two words were completely unrelated or inconsistent.

Finally, for incidental memory recall, there was one measured variable: Accuracy (coded as a binary variable, 1 – correct vs. 0 – incorrect), i.e., whether or not participants were able to accurately recall (cued recall test) or select (2AFC test) the unrelated word presented during the exposure phase.

The data from the four tests (relatedness judgments, associate production, cued recall, and 2AFC) were analysed by fitting mixed-effects models using the *lme4* package (Bates et al., 2014) implemented in R (version 4.2.2, R Core Team, 2022). For the analysis of the accuracy data for semantic relatedness judgments and incidental memory tests, binomial generalised linear mixed-effects models were fitted to the trial-level data, with correct responses coded as 1 and incorrect responses coded as 0. For the analysis of reaction time data (in ms) from the speeded semantic relatedness judgement task and for the associate production task data (continuous consistency measure from 0 to 1), linear mixed-effects models were used.

To address our hypotheses, the following hypothesis matrix was used, from which custom contrasts were derived via the generalised inverse for inclusion in the mixed effects models (see Schad et al., 2020). This hypothesis matrix allowed us to test our hypotheses regarding the fixed effect of Priming, which had four levels (Expected, Neutral, Unexpected, Unprimed). The *All Primed vs. Unprimed* contrast assesses whether or not there is an overall word-meaning priming effect, while the *Expected vs. Neutral* and the *Unexpected vs. Neutral* contrasts test for an effect of expectedness:

*All Primed s vs. Unprimed:* [Expected = 1, Neutral = 1, Unexpected = 1, Unprimed = –3]

*Expected vs. Neutral:* [Expected = 1, Neutral = –1, Unexpected = 0, Unprimed = 0]

*Unexpected vs. Neutral:* [Expected = 0, Neutral = –1, Unexpected = 1, Unprimed = 0]

This resulted in the following contrasts in the contrast matrix (derived using the generalised inverse with the *ginv2* function in R; Schad et al., 2020):

*All Primed vs. Unprimed:* [Expected = 0.083, Neutral = 0.083, Unexpected = 0.083, Unprimed = –0.25]

*Expected vs. Neutral:* [Expected = 0.667, Neutral = –0.333, Unexpected = –0.333, Unprimed = 0]

*Unexpected vs. Neutral:* [Expected = –0.333, Neutral = –0.333, Unexpected = 0.667, Unprimed = 0]

Each model contained the random effects of Participant and Item. The *buildmer* package (Voeten, 2021) was used to determine the most parsimonious random effects structure, starting out with the most complex structure and then identifying the most parsimonious model to successfully converge via stepwise elimination of random effects (Barr et al., 2013). The ‘bobyqa’ optimiser was used to increase the likelihood of convergence. In the linear mixed-effects model, if the residuals were not normally distributed (determined via Q-Q plots), the response time data were log-transformed.

### 2.2 Results

#### 2.2.1 Relatedness judgements

##### 2.2.1.1 Accuracy

**Figure 2:**
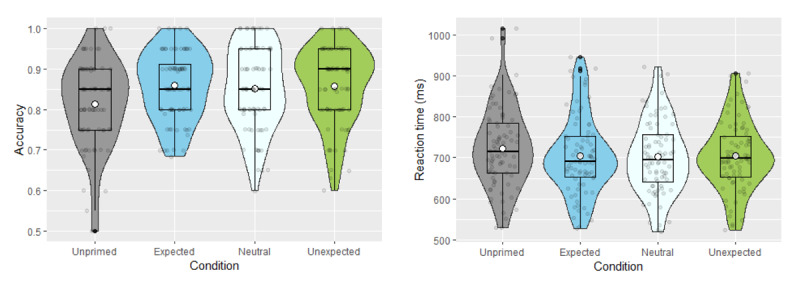
Accuracy (left) and reaction times (right) in the relatedness judgement task (Exp. 1). White dots represent the mean, while horizontal black bars represent the median for each condition. The black boxplots indicate the interquartile range and the density functions represent the distribution of participant means, with individual scores shaded in grey.

As summarised in Figure 2 (left), mean accuracy rates were 85.88% (*SD* = 8.42%) in the expected condition, 85.10% (*SD* = 10.14%) in the neutral condition, 85.71% (*SD* = 9.74%) in the unexpected condition, and 81.35% (*SD* = 10.78%) in the unprimed condition. Contrary to our predictions, the mixed-effect model revealed a non-significant word-meaning priming effect (β = 1.153, *SE* = 0.650, *z* = 1.776, *p* = .076), and there was also no significant effect of expectancy on performance (*p*s > .544).

##### 2.2.1.2 Reaction times

Mean reaction times in the different conditions were as follows: Expected, 692.87ms (*SD* = 90.21ms); neutral, 692.55ms (*SD* = 88.50ms); unexpected, 690.37ms (*SD* = 82.36ms); unprimed, 707.29ms (*SD* = 97.94ms). Consistent with the accuracy data, the reaction time data also showed a nonsignificant trend towards a word-meaning priming effect (all primed conditions vs. unprimed: β = –0.060, *SE* = 0.043, *z* = –1.379, *p* = .168), and there was no evidence of an expectedness effect (*p*s > .724).

##### 2.2.1.3 Rate of correct responses (RCR)

**Figure 3:**
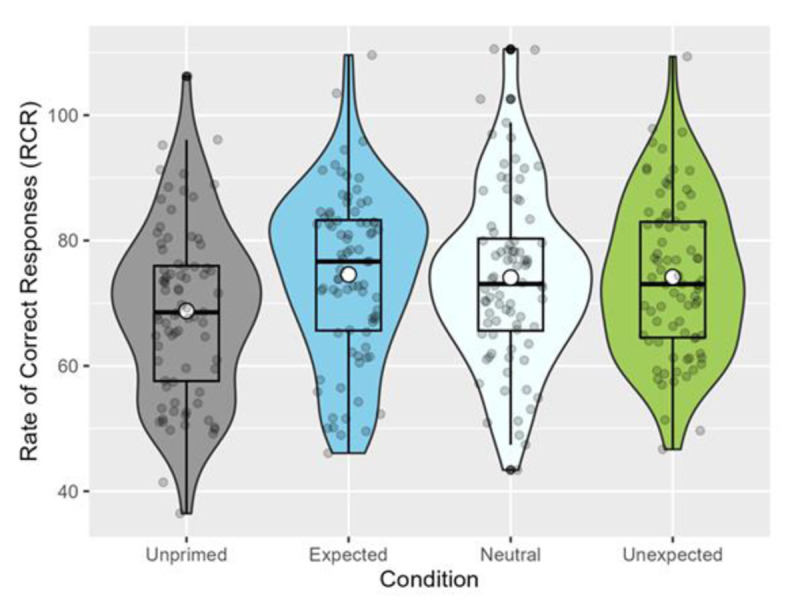
Rate of correct responses (RCR) for the relatedness judgement task (Exp. 1). White dots represent the mean, while horizontal black bars represent the median for each condition. The black boxplots indicate the interquartile range and the density functions represent the distribution of participant means, with individual scores shaded in grey.

As an exploratory analysis, we combined the accuracy and reaction time data to form a measure of the rate of correct responses (RCR) a participant makes in this task, defined as the (proportion of correct responses/((RT in ms/60,000)) (see Vandierendonck, 2017, for an evaluation of this method). This has been found to be a more sensitive measure of priming (compared to using either accuracy or reaction times alone), and is used frequently in semantic priming studies (e.g., Was et al., 2019). Note that since the RCR measure consists of one RCR score per participant, the lack of trial-level information necessitates a switch from mixed-effects models to ANOVAs. We submitted the RCR values to an ANOVA, which revealed a significant effect of condition, *F*(3, 316) = 3.379, *p* = .019. Following up on this, multiple comparisons using Dunnett contrasts showed that all three of the exposure conditions differed significantly from the unprimed condition (expected vs. unprimed, *p* = .018; neutral vs. unprimed, *p* = .035; unexpected vs. unprimed, *p* = .031; see Figure 3 below).

#### 2.2.2 Associate production

##### 2.2.2.1 Word2vec similarity analysis

Following our pre-registration, we analysed the associate production data using word2vec similarity analysis. We computed cosine similarity values between participants’ responses to target words and the corresponding probe word (the primed meaning aspect). The mean cosine similarity scores for each condition are as follows: Expected, 0.288 (*SD* = 0.202), neutral, 0.295 (*SD* = 0.198), unexpected, 0.279 (*SD* = 0.208), unprimed, 0.261 (*SD* = 0.180). There was neither an overall priming (β = 0.072, *SE* = 0.061, *z* = 1.176, *p* = .240), nor an expectedness effect (*p*s > .652).

##### 2.2.2.2 Semantic similarity (human raters)

As an exploratory analysis and following prior studies on word-meaning priming (e.g., Curtis et al., 2022; Gaskell et al., 2019), we additionally indexed consistency via human judgement. We recruited an independent sample of participants (*n* = 124) to rate the semantic similarity between the associate production responses and the primed meaning aspect (on a scale of 1 to 10). Each participant rated a subset ranging between 209 to 266 word pairs (depending on how many unique responses were provided for each probe word). Consistent with the analysis using cosine similarity derived from word2vec, we did not observe a word-meaning priming effect using this measure (*p* = .130), nor an effect of expectedness (*p*s > .829). The mean scores by condition are as follows: Expected, 5.245 (*SD* = 1.29), neutral, 5.263 (*SD* = 1.472), unexpected, 5.215 (*SD* = 1.50), unprimed, 4.89 (*SD* = 1.39).

#### 2.2.3 Incidental memory

##### 2.2.3.1 Cued recall

The cued recall task showed no effect of condition either (*p*s >.598), with most participants performing at floor, suggesting that this task was too difficult.

##### 2.2.3.2 2AFC

Following our pre-registered analysis, one-sample *t*-tests showed that participants performed above chance in all three conditions in the 2AFC task: Expected, 59.38% (*SD* = 15.10%), neutral, 60.31% (*SD* = 13.18%), unexpected, 61.56% (*SD* = 15.13%) (*p*s <.001). However, contrary to our prediction, there were no significant effects of expectedness (*p*s > .349).

### 2.3 Discussion

Contrary to our predictions, the results of Experiment 1 showed no evidence of a predictability effect on either the strength of word-meaning priming or performance in the incidental memory tests. However, the overall priming effect was not significant in our pre-registered measures and was only significant when using an exploratory measure combining accuracy and reaction times for the relatedness judgement task. Therefore, one explanation for the lack of predictability effects is that the current experimental procedure might not have been ideally suited to elicit strong priming effects overall, making it difficult to observe what are likely to be more subtle effects of predictability on the strength of priming. On the other hand, the issue might be that participants were not required to actively predict the sentence-final target word, and its presentation together with the rest of the sentence may have been enough to hinder any predictability effect. To further investigate these possibilities, Experiment 2 used a modified design with the goal of improving our chance in detecting possible predictability effects as well as observing more robust word-meaning priming.

## 3. Experiment 2

Experiment 2, which was pre-registered prior to data collection (https://osf.io/8q5tz), followed the same design as Experiment 1, with the exception of the following key modifications:

– There was a 5000 ms delay between the presentation of the lead-in sentence and the sentence-final target word, enhancing the opportunity for participants to predict the final word.
– Participants were asked to rate how surprised they were upon encountering the sentence-final target word (on a scale of 1–10) after every trial of the exposure phase. This was intended both to encourage participants to attend to whether the ending was unexpected and to allow us to verify that the experimental materials differed in the surprisal they elicited (e.g., that the unexpected endings elicit more surprisal than the expected and neutral endings, etc.).

As explained in the Introduction, if word-meaning priming is affected by similar factors as have been shown to affect memory in (other) episodic memory paradigms (e.g., Quent et al., 2022), then the strength of the word-meaning priming effect should be affected by the relative expectedness of the target word, with both highly expected as well as highly unexpected items leading to stronger word-meaning priming effects. Therefore, the central question remained whether expectedness had an impact on word-meaning priming. As in Experiment 1, we also tested the prediction derived from the models of episodic memory cited above (e.g., SLIMM) that memory for incidental aspects of the experiment would be better if these are processed in close temporal proximity to the unexpected input (compared to expected or neutral input). A 2AFC test probing memory for incidental aspects of the main experiment addressed this hypothesis. We dropped cued recall in Experiment 2 due to low performance (floor effects) in the previous experiment.

### 3.1 Methods

#### 3.1.1 Participants

Ninety-two participants, aged between 18 and 35 years, were recruited online via Prolific and paid at the rate of £7 per hour. The recruitment and exclusion criteria were the same as for Experiment 1. We had to discard the data from 11 participants in total (six due to their performance in the relatedness judgement task, four due to failing the comprehension questions in the filler task, and one who failed both of the above), resulting in a final dataset of 81 participants (41 female; mean age = 28.22, *SD* = 4.49)

#### 3.1.2 Materials

The materials were the same as used for Experiment 1, except that four target items (along with their sentence triplets) were removed as they elicited the weakest priming effect in Experiment 1. This left us with 76 items overall.

#### 3.1.3 Procedure

The procedure remained the same as for Experiment 1, except that in Experiment 2, each sentence was first presented without the sentence-final target word, which appeared after a delay of 5s underneath the lead-in sentence in the centre of the screen. The delay was implemented to ensure participants fully comprehended the preceding sentence and to allow them to build up a prediction for the sentence-final word, as they were instructed to do.The sentence remained on the screen when the target word was presented. After the target word appeared, participants were asked to rate how surprised they were to encounter that word (on a scale of 1 to 10). All other tasks remained the same (though note that there was no cued recall task in Experiment 2 due to low performance in the first experiment).

#### 3.1.4 Analysis

Most aspects of the analyses remain the same as in Experiment 1, with one exception being that we pre-registered the RCR measure as our primary analysis for relatedness judgement. However, we also pre-registered and conducted exploratory analyses using accuracy and reaction time separately in two independent mixed-effect models (for comparison with prior studies). For associate production, we decided to drop the *word2vec* analysis and instead used continuous consistency ratings done by third-party raters as our pre-registered measure. Additionally, we ran an exploratory analysis using a binary rating scale (not pre-registered).

### 3.2 Results

#### 3.2.1 Relatedness judgements

##### 3.2.1.1 Rate of correct responses (RCR)

**Figure 4:**
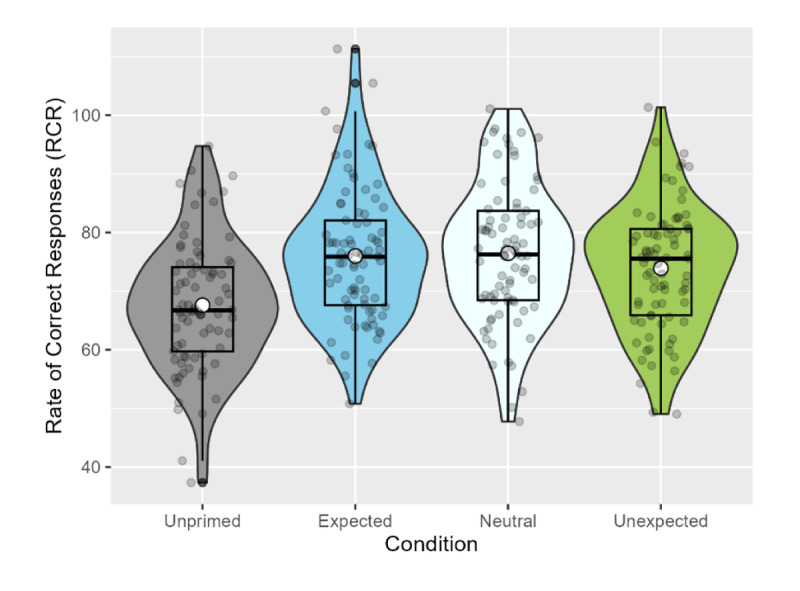
Rate of correct responses (RCR) in the relatedness judgement task (Exp. 2). White dots represent the mean, while horizontal black bars represent the median for each condition. The black box plots indicate the interquartile range and the density functions represent the distribution of participant means, with individual scores shaded in grey.

For our confirmatory analysis, we again used the rate of correct responses (RCR) measure to assess participants’ performance in relatedness judgement. An ANOVA revealed a significant effect of condition, *F*(3, 320) = 10.65, *p* < .001. Multiple comparisons using Dunnett contrasts showed that all three of the exposure conditions differed significantly from the unprimed condition (expected vs. unprimed, *p* < .001; neutral vs. unprimed, *p* < .001; unexpected vs. unprimed, *p* = .001). The mean RCR values by condition were as follows (see Figure 4): Expected, 75.99 (*SD* = 11.54), neutral, 76.46 (*SD* = 11.81), unexpected, 73.90 (*SD* = 10.64), unprimed, 67.59 (*SD* = 11.02).

##### 3.2.1.2 Accuracy

**Figure 5:**
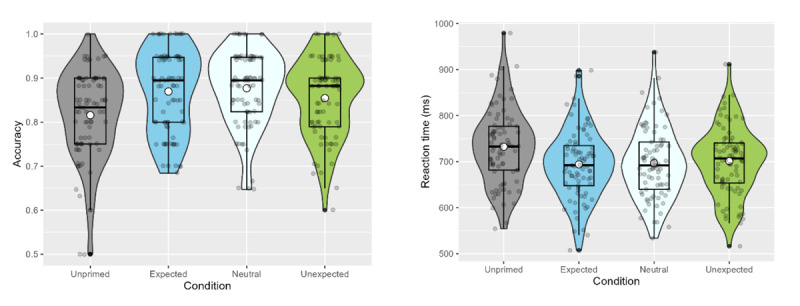
Mean accuracy and reaction times in the relatedness judgement task (Exp. 2). White dots represent the mean, while horizontal black bars represent the median for each condition. The black box plots indicate the interquartile range and the density functions represent the distribution of participant means, with individual scores shaded in grey.

Analysis of the relatedness judgement accuracy data showed a significant word-meaning priming effect (β = 1.378, *SE* = 0.621, *z* = 2.220, *p* = .026), but no effect of expectancy on performance (*p*s > .447). The mean accuracy rates were 86.96% (*SD* = 9.35%) in the expected condition, 87.69% (*SD* = 8.62%) in the neutral condition, 85.45% (*SD* = 8.96%) in the unexpected condition, and 81.57% (*SD* = 10.01%) in the unprimed condition (see Figure 5).

##### 3.2.1.3 Reaction times

The reaction time data also showed an overall word-meaning priming effect (all primed conditions vs. unprimed: β = –0.188, *SE* = 0.047, *z* = –4.053, *p* = <.001), but again, there was no evidence of a predictability effect (*p*s > .269). Mean reaction times in the different conditions were as follows: Expected, 683.672ms (*SD* = 81.078ms); neutral, 679.553ms (*SD* = 81.822ms); unexpected, 687.413ms (*SD* = 75.382ms); unprimed, 719.835ms (*SD* = 85.323ms).

#### 3.2.2 Associate production

##### 3.2.2.1 Continuous similarity ratings

As in Experiment 1 (although now pre-registered), participants’ associate production responses were rated by an independent sample of Prolific participants (*n* = 24) as to how closely related they were in meaning to the primed meaning aspect (relatedness judgement probe). There was a non-significant effect in the predicted direction with regard to overall word-meaning priming (all primed conditions vs. unprimed, β = 0.674, *SE* = 0.557, *z* = 1.246, *p* = .213), but no effect of expectedness. The mean similarity rating for the expected condition was 5.379 (*SD* = 1.469), 5.668 (*SD* = 1.473) for the neutral condition, 5.396 (*SD* = 1.402) for the unexpected condition, and 5.258 (*SD* = 1.341) for the unprimed condition.

##### 3.2.2.2 Binary ratings

As an exploratory analysis (not pre-registered), the word pairs (consisting of participants’ associate production responses and the corresponding relatedness judgement probe word) were rated on a binary scale by the experimenter who was blind to the condition of the items. It was judged whether a word pair was semantically related (1) or not (0), i.e., whether or not the response provided by participants in the associate production task was consistent with the primed meaning aspect. This analysis revealed a strong word-meaning priming effect (β = 1.612, *SE* = 0.482, *z* = 3.344, *p* < .001), but no expectedness effect (*p*s > .202).

To ensure that these rating results were not influenced by the experimenter’s familiarity with the experimental stimuli or any other individual factors, all the items were rated by an additional rater from outside the research team, using the same binary scale and following the same guidelines. Using only the items were both raters were in agreement (93.38% of the data) showed the same pattern of results, again revealing a word meaning priming effect (β = 0.237, *SE* = 0.081, *z* = 2.918, *p* = .004), but no effect of expectedness (*p*s > .360).

#### 3.2.3 Incidental memory

##### 3.2.3.1 2AFC

Participants performed above chance in all three conditions (*p*s < .001, by one-sample t-tests), with a mean accuracy of 58.97% (SD = 13.78%) in the expected condition, 57.54% (SD = 12.70%) in the neutral condition, and 59.86% (SD = 14.55%) in the unexpected condition. Similar to the results from Experiment 1, there was no effect of expectedness on incidental memory performance as assessed by the 2AFC task (*p*s > .576).

### 3.3 Discussion

As in Experiment 1, the results of Experiment 2 showed no evidence of a predictability effect on word-meaning priming. Likewise, the relative expectedness of the target word did not influence participants’ performance in the incidental memory test either. In contrast to Experiment 1, however, the overall priming effect was robust across all but one of the tasks assessing priming. While the continuous similarity ratings of the associate production task did not reveal any effects, the binary ratings completed by two independent experimenters showed a strong priming effect. Note that using binary ratings (completed either by the experimenters as here or in Gaskell et al., 2019, or Rodd et al., 2013, or by the participants as in Rodd et al., 2016) is the norm in word-meaning priming studies, though it is important to acknowledge that it is possible that dichotomising a continuous variable may reduce statistical power and could inflate Type I error rates, which could potentially lead to misleading conclusions (Maxwell & Delaney, 1993). However, in combination with the RCR results from the relatedness judgement task, which showed a strong priming effect, we think that there is good evidence for the existence of a word-meaning priming effect in Experiment 2.

While the design changes in Experiment 2 seem to have produced a stronger word-meaning priming effect, there was no evidence of a predictability effect in any task. Speculatively, one reason for the absence of expectedness effects might be that the previous two experiments did not include a delay interval during which the episodic memory trace established during the exposure phase could be consolidated. In other words, perhaps the influence of predictability on word-meaning priming is too subtle to be observed at immediate test, but may emerge after an opportunity for offline memory consolidation (e.g., during overnight sleep; Rasch & Born, 2013). Therefore, highly unexpected target words (which are presumed to be maximally reliant on the hippocampus for encoding due to the predictions errors experienced when they are processed; van Kesteren et al., 2013) might show stronger word-meaning priming effects than neutral target words after a delay interval including sleep, but not necessarily at immediate test or after a delay filled with wakefulness. We therefore ran a third experiment using a sleep/wake design with a 12-hour consolidation interval.

## 4. Experiment 3

Experiment 3 (https://osf.io/8nw6k) was intended to follow up on the previous two experiments and to examine the effects of semantic predictability and sleep on word-meaning priming and incidental memory. The design and procedure for Experiment 3 was the same as for Experiment 2, except for the following changes:

– There was a 12-hour delay between the exposure and the test phases, which were separated either by overnight sleep or wakefulness. Half of the participants completed the exposure phase between 8–10am in the morning and the tests between 8–10pm at night (Wake group), whereas the other half completed the exposure phase between 8–10pm at night and did the tests the following morning between 8–10am (Sleep group). Note that it was no longer necessary to include a filler task after the exposure phase due to the longer delay.
– The number of within-subjects conditions was reduced to three, retaining the Neutral, Unexpected, Unprimed conditions. We did not include an Expected condition i) to increase power, ii) because the Expected and Neutral conditions showed a comparable pattern of results in previous experiments, and iii) because the critical prediction with regard to an influence of sleep on how expectedness may affect word-meaning priming concerned the Unexpected condition (but not necessarily the Expected condition; van Kesteren et al., 2013).

Using the same reasoning as for the two previous experiments, if word-meaning priming is affected by factors that have been shown to affect memory in (other) episodic memory paradigms, then the strength of the word-meaning priming effect should be affected by the relative expectedness of the target word. Moreover, we expected these effects to be larger after a delay interval including sleep, due to the increased opportunity for hippocampal memory consolidation during sleep, which should strengthen the episodic memory traces that are assumed to be encoded during sentence exposure.

As in Experiments 1 and 2, we again tested whether memory for incidental aspects of the experiment would be better if these are processed in close temporal proximity to unexpected input (compared to neutral input). We used the same 2AFC test from Experiments 1 and 2. Again, we expected these effects to be stronger after sleep, assuming that they would rely on hippocampal memory traces.

### 4.1 Methods

#### 4.1.1 Participants

The exclusion criteria were the same as for Experiments 1 and 2, but our power analysis (based on Brysbaert & Stevens, 2018) indicated that we needed 60 participants in each group to ensure our mixed-effects modelling approach was adequately powered (resulting in 120 participants overall). We recruited participants via Prolific using a random allocation approach (i.e., participants signed up for the study and were then randomly allocated to either the Sleep or the Wake group; see Mak, 2024). We recruited participants until we reached 60 complete datasets per group, along the way excluding participants who did not come back to the second part during the specified time frame, or who started the first part outside of that time frame. Of those who completed both parts at the designated times, we discarded 12 datasets (all due to their performance in the relatedness judgement task; recall that there was no filler task in this design, hence there were no exclusions due to failing the comprehension questions associated with that task, cf. Exp. 1 and 2). Our final sample consisted of 60 participants in the Sleep group (35 female; mean age = 25.08, *SD* = 3.92) and 60 participants in the Wake group (38 female; mean age = 24.7, *SD* = 4.61).

#### 4.1.2 Materials

The materials were the same as those in Experiment 2, except that one further item was removed in order to fully balance the allocation of items to lists in this new design. There were thus 75 items (with 25 in each of the Neutral, Unexpected, and Unprimed conditions).

#### 4.1.3 Design

Experiment 3 consisted of a mixed design with one within-subjects factor (Priming) which had three levels (Neutral, Unexpected, Unprimed), and one between-subjects factor (Delay) which had two levels (overnight sleep vs. daytime wakefulness).

#### 4.1.4 Procedure

This online experiment was split into two parts (exposure and test phases) which were separated by 12 hours either spent awake or asleep. Those in the Wake group completed the sentence exposure phase in the morning (8–10 AM) and returned to complete the test phase (consisting of the relatedness judgement task, associate production task, and incidental memory tests) in the evening (8–10 PM) on the same day, whereas the other half of the participants completed the sentence exposure phase in the evening (8–10 PM) and returned to complete the tests the following morning (8–10 AM). Note that due to the online nature of this experiment, all participants completed the study unsupervised and at a location of their own choosing. Despite this, prior work from our group has used this online procedure, successfully detecting sleep-related memory effects, such as a post-sleep benefit in free (Mak, O’Hagan, Horner & Gaskell, 2023) and cued recall (Ashton & Cairney, 2021; Mak, Curtis, Rodd, & Gaskell, 2024).

#### 4.1.5 Analysis

As in the previous experiments, the RCR measure was analysed using an ANOVA, and where appropriate, Dunnett contrasts were run to compare each of the primed conditions to the unprimed condition. Associate production and incidental memory (2AFC) were analysed using binomial generalised linear mixed-effects models with correct responses coded as 1 and incorrect responses coded as 0. A mixed-effects logistic analysis was run using a model with one effect-coded between-participants independent variable, Group (Sleep vs. Wake). Within participants, the independent variable Priming had three levels (Neutral, Unexpected, Unprimed), coded using orthogonal Helmert contrasts to compare (i) the Unprimed condition vs. the two Primed conditions (OverallPriming), and (ii) the two primed conditions with each other (BetweenPriming). The model building process was the same as in Experiments 1 and 2.

### 4.2 Results

#### 4.2.1 Relatedness judgements

##### 4.2.1.1 Rate of correct responses (RCR)

The RCR values were as follows: Sleep – neutral, 73.60 (*SD* = 13.96); sleep – unexpected, 72.64 (*SD* = 12.77); sleep – unprimed, 70.39 (*SD* = 12.17); wake – neutral, 72.75 (*SD* = 13.71); wake – unexpected, 71.79 (*SD* = 13.29); wake – unprimed, 72.30 (*SD* = 13.37). Submitting these RCR values to a mixed 3 (Priming) x 2 (Group) ANOVA yielded no significant effects or interactions (all *p*s > .318), suggesting that in the relatedness judgement task, there were no significant differences between the different priming conditions (based on the level of expectedness) and the two groups (sleep vs. wake).

##### 4.2.1.2 Accuracy

In a pre-registered subsidiary analysis, we looked at the accuracy and response times separately (for comparability with previous work). Mean accuracy scores across the groups and conditions were as follows: Sleep – neutral, 86.82%; sleep – unexpected, 86.11%; sleep – unprimed, 83.17%; wake – neutral, 84.88%; wake – unexpected, 84.86%; wake, unprimed, 82.75%. A mixed-effects analysis showed no significant effects or interactions, consistent with the RCR analysis.

##### 4.2.1.3 Reaction times

Participants’ mean reaction times in the relatedness judgement task were as follows: Sleep – neutral, 701.22ms; sleep – unexpected, 722.63ms; sleep – unprimed, 725.75ms; wake – neutral, 708.49ms; wake – unexpected, 710.20ms; wake – unprimed, 721.16ms. A mixed-effects analysis revealed a small but significant effect of Priming (neutral and unexpected vs. unprimed), with the primed conditions showing faster reaction times than the unprimed condition (β = 0.013, *SE* = 0.006, *z* = 2.349, *p* = .018). There were no other significant effects or interactions.

#### 4.2.2 Associate production

##### 4.2.2.1 Binary ratings

The means for the binary ratings of the associate production responses were as follows: Sleep – neutral, 45%; sleep – unexpected, 44%; sleep – unprimed, 39%; wake – neutral, 47%; wake – unexpected, 45%; wake – unprimed, 37%. A mixed-effects analysis showed a marginally non-significant effect of Priming (neutral and unexpected vs. unprimed), with the primed conditions showing higher consistency ratings than the unprimed condition (β = 0.098, *SE* = 0.056, *z* = 1.762, *p* = .078). However, this effect was qualified by a significant interaction of Priming and Group, with greater priming in the Wake than the Sleep group (β = 0.090, *SE* = 0.043, *z* = 2.102, *p* = .034). Follow-up comparisons using the *emmeans* package revealed a significant difference between the Neutral and Unprimed conditions in the Wake group only (*p* = .015; adjusted for multiple comparisons using the Tukey method). There were no other significant effects.

#### 4.2.3 Incidental memory

##### 4.2.3.1 2AFC

The mean accuracy values were as follows: Sleep – neutral, 51.73% (*SD* = 12.63%); sleep – unexpected, 51.50% (*SD* = 13.11%); wake – neutral, 54.34% (*SD* = 11.64%); wake – unexpected, 55.19% (*SD* = 14.24%). The mixed-effects model using the 2AFC task data yielded no significant effects or interactions (all *p*s > .458), and follow-up analyses revealed that none of the conditions was above chance by one-sample t-test (all *p*s > .271). The chance performance in this experiment is in contrast to the above-chance performance in experiments 1 and 2, suggesting that memory for these incidental details decays rather rapidly, being only observable after a short delay of about 30 minutes but not after a 12-hour interval.

### 4.3 Discussion

The results of Experiment 3 showed a small word-meaning priming effect in the RT data from the relatedness judgement task (with differences between the primed and unprimed conditions), but these differences did not emerge in the accuracy data or the combined RCR measure (which was the primary, pre-registered outcome measure). There was no effect of predictability in any of the three measures. The associate production task showed effects of priming in both groups (Sleep and Wake), but again none of the hypothesised effects of predictability emerged. Memory performance in the 2AFC task was at chance in all conditions, with no differences between the conditions or groups.

Experiment 3 is thus the third experiment to show no evidence of predictability effects on word-meaning priming or incidental memory across a range of tasks and measures. One final possibility to test is whether this absence of expectedness effects was due to our sentences being semantically congruent (even though the sentence-final words are highly unexpected and the overall meaning of the sentences is surprising or odd). It is possible that predictability effects only emerge when the linguistic input is sufficiently unexpected to the point of being semantically incongruent. Experiment 4 was designed to test this possibility by assessing whether we would observe effects of predictability on incidental memory if the sentence-final target word is semantically incongruent with the rest of the sentence (i.e., if incongruency is required to observe a prediction error effect). Note that word-meaning priming can no longer be tested with this new incongruent condition because having an incongruent sentence ending changes the meaning of the sentence and results in the critical sentences no longer ending in the same final word. This means that the critical comparison between the effects of encountering a certain word in a specific context vs. not encountering it at all becomes impossible, since the sentence-final word in each sentence group will be different depending on which condition it appears in. While with this design we are no longer able to assess word-meaning priming, we reasoned that this should be a blunter measure to elicit predictability effects, considering that all previous studies showing effects of predictability have used rather simple memory tests (rather than semantic judgements as used in the tests assessing word-meaning priming).

## 5. Experiment 4

Experiment 4 (https://osf.io/tg8fp) was an incidental memory study that included an additional ‘incongruent’ condition, featuring sentences in which the sentence-final word was semantically incongruent with the preceding context. (e.g., “While Hannah enjoyed the symphony orchestra’s performance, she was concerned about the fact that her ears were still ringing from last night’s spectacular *pumpkin*.”). These incongruent sentences were constructed by taking all but the final word of the expected sentences and adding an incongruent ending, with the incongruent and expected/neutral/unexpected sentence-final words matched on a number of variables known to affect linguistic processing (number of letters, lexical frequency, imageability, and concreteness). The unexpected condition describes events that are unlikely to occur in real life, but these events do not violate any laws of physics or common sense (e.g., “Each Bonfire Night, Hannah is concerned about the ear-piercing bangs caused by the spectacular *concert*.”). In contrast, sentences in the incongruent condition describe events that are highly unrealistic and difficult to picture (i.e., it is very difficult to imagine a scenario in which a pumpkin could somehow cause someone’s ears to ring for several hours). Memory was assessed a) for the sentence-final word using an old/new recognition test as well as b) for the adjective immediately preceding the sentence-final word using a cued recall test, which was designed to test memory for contextual details surrounding the expected/neutral/unexpected/incongruent sentence ending.

### 5.1 Methods

#### 5.1.1 Participants

The recruitment criteria were the same as for Experiments 1 to 3. No datasets were excluded based on our pre-registered exclusion criteria. Our final sample, recruited via Prolific, consisted of 80 participants as pre-registered (37 female; mean age = 28.83, SD = 4.24).

#### 5.1.2 Materials

The materials for Experiment 4 consisted of 72 items from Experiment 3, plus an additional Incongruent condition (as explained above). The stimuli from the previous experiments were also amended slightly so that all the sentences in what used to be an item triplet (now quartet) contained the same adjective immediately preceding the sentence-final word. This was necessary to ensure that the cued recall memory test (described below) was not biased in terms of some items potentially being easier to recall than others.

#### 5.1.3 Procedure

Participants first completed the sentence exposure phase (as in Experiments 2 and 3) as well as the short filler task (as described for Experiment 1). After that, they moved onto the test phases. They first completed an old/new recognition test, where they were presented with the 72 target words from the exposure phase as well as 72 unseen words (36 of which were lure, i.e., the words that were expected but not encountered in the Unexpected and Incongruent conditions; and the other 36 being control words that were not predicted and that were unrelated to any of the encountered or expected words). Participants were asked to judge whether they recognised the word from the exposure phase by pressing a button on their keyboard (F = Old/Recognise, J = New/Don’t recognise). They were given unlimited time to respond and moved onto the next trial once they submitted their response.

The second memory test consisted of a fill-in-the-blank task (cued recall), where participants were presented with the 72 sentences from the exposure phase, with the adjective preceding the sentence-final word blanked out. They were asked to recall that adjective by typing it in, again with no time limit. They moved on to the next trial by submitting their response using the “Return” key.

#### 5.1.4 Analysis

For the recognition test, there was one measured variable: Accuracy (coded as a binary variable, 1 – correct vs. 0 – incorrect), i.e., whether or not participants were able to correctly accept or reject the test words depending on whether or not they have encountered them in the exposure phase (i.e., to correctly rate them as ‘old’ or ‘new’). For incidental memory recall (fill-in-the-blank), there was also one measured variable: Accuracy (coded as a binary variable, 1 – correct vs. 0 – incorrect), i.e., whether or not participants are able to accurately recall the unrelated adjective that preceded the sentence-final word presented during the exposure phase. The models were fitted as explained in the preceding experiments above.

To address our hypothesis regarding the pattern of performance expected in the recognition test, where we expected both the Expected as well as the Unexpected and Incongruent conditions to show better memory than the Neutral condition (i.e., we predicted a U-shaped function of memory against expectedness; Quent et al., 2022), we used treatment coding using the Neutral condition as the baseline, to which all three other conditions were compared. The Expected-Neutral, Unexpected-Neutral, and Incongruent-Neutral contrasts would have to be significant (in the predicted direction) in order for the results to constitute evidence for the experimental hypothesis. As a subsidiary analysis, we pre-registered to use the *emmeans* package to compare the Unexpected and the Incongruent conditions with each other.

For cued recall (fill-in-the-blank), we predicted better memory for contextual details that were processed in close proximity to unexpected and incongruent sentence-final words (i.e., a prediction error-driven memory boost, as hypothesised by the SLIMM model; van Kesteren et al., 2013). Therefore, we wanted to separately compare both the Unexpected and the Incongruent conditions to the combined Neutral and Expected conditions (no difference was expected between these latter two conditions, hence why they were pooled to minimise the number of comparisons made). Additionally, we wanted to compare the Unexpected and Incongruent conditions with each other to test for potential differences between them (there was no hypothesis as to how these might differ). If at least one of the two Neutral+Expected vs. Unexpected/Incongruent contrasts is significant, this would constitute evidence for the experimental hypothesis.

The following hypothesis matrix was used, from which custom contrasts were derived via the generalised inverse for inclusion in the mixed effects models (see Schad et al., 2020):

Coefficients in the hypothesis matrix for the incidental memory test:

- *Neutral and Expected vs. Unexpected:*
  [Expected = –1, Neutral = –1, Unexpected = 2, Incongruent = 0]
- *Neutral and Expected vs. Incongruent:*
  [Expected = –1, Neutral = –1, Unexpected = 0, Incongruent = 2]
- *Unexpected vs. Incongruent:*
  [Expected = 0, Neutral = 0, Unexpected = –1, Incongruent = 1]

This resulted in the following contrasts in the contrast matrix (derived using the generalised inverse with the *ginv2* function in R; Schad et al., 2020):

- *Neutral and Expected vs. Unexpected:*
  [Expected = –0.125, Neutral = –0.125, Unexpected = 0.292, Incongruent = –0.042]
- *Neutral and Expected vs. Incongruent:*
  [Expected = –0.125, Neutral = –0.125, Unexpected = –0.042, Incongruent = 0.292]
- *Unexpected vs. Incongruent:*
  [Expected = 0, Neutral = 0, Unexpected = –0.167, Incongruent = 0.167]

As for the previous experiments, each model contained the random effects of Participant and Item, with the most parsimonious random effects structure determined via *buildmer* (Voeten, 2021).

### 5.2 Results

#### 5.2.1 Old/new recognition

**Figure 6:**
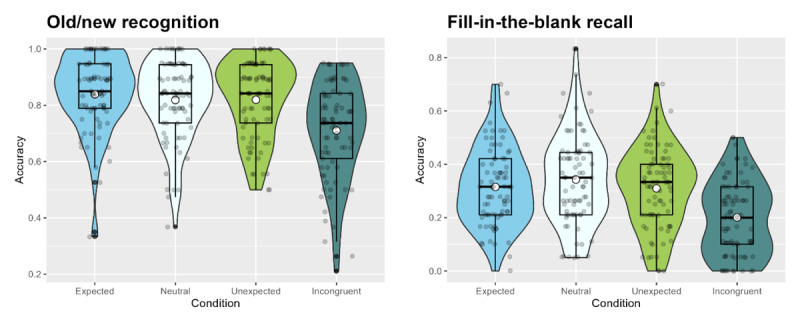
Accuracy in the old/new recognition task (left) and the cued recall task (right) in Exp. 4. White dots represent the mean, while horizontal black bars represent the median for each condition. The black boxplots indicate the interquartile range and the density functions represent the distribution of participant means, with individual scores shaded in grey.

Performance in the old/new recognition memory test is shown in Figure 6 below (left panel). The mean accuracy values for each condition were as follows: Expected, 81.85% (*SD* = 14.10%); Neutral, 83.88% (*SD* = 13.94%); Unexpected, 81.98% (*SD* = 3.67%); Incongruent, 71.01% (*SD* = 13.67%). The mixed-effects model revealed a significant difference between the neutral and the incongruent conditions (β = 0.745, *SE* = 0.153, *z* = 4.876, *p* <.001), but not between the neutral baseline and either of the other conditions (*p*s > .258). However, notably, this effect was in the opposite direction to our hypothesis, with the incongruent words being recognised significantly *less* accurately than the neutral ones.

#### 5.2.2 Fill-in-the-blank (cued recall)

Participants’ performance in the fill-in-the-blank task (cued recall) is shown in the right panel of Figure 6. The mean scores for each condition were as follows: Expected, 31.51% (*SD* = 14.46%); Neutral, 34.28% (*SD* = 16.85%); Unexpected, 30.95% (*SD* = 15.19%); Incongruent, 20.07% (*SD* = 13.88%). As in the recognition test, the incongruent condition showed significantly lower mean values compared to the neutral condition (β = 1.065, *SE* = 0.285, *z* = 3.742, *p* <.001), again contrary to our hypothesis derived from current models of episodic memory.

### 5.3 Discussion

Experiment 4 showed that, contrary to our hypotheses, there was no memory benefit for either the Expected, Unexpected or Incongruent condition. In fact, items presented in the Incongruent condition were remembered significantly *worse* than those presented in the other three conditions (which did not show any between-condition differences). This result is perhaps less surprising in the fill-in-the-blank recall test, where the Expected, Neutral, and to a lesser extent also the Unexpected condition could have benefitted from context at test by providing a more reasonable basis for guessing as to what the missing adjective was. It would have been harder to do so in the Incongruent condition, and this alternative explanation may partially account for the pattern seen in the recall test.

In the old/new recognition test, however, an explanation invoking a benefit of context at test is not applicable, as the critical sentence-final words as well as the foils were presented one-by-one without any context. It is therefore reasonable to assume that in the Incongruent condition, encoding was impaired, rather than the effect simply being due to a lesser benefit from context at test. One highly speculative explanation for this is that the lack of plausibility in the Incongruent condition may have led the language system to essentially consider the final word as a mistake, or in any case less useful information with regard to future comprehension demands. It might not have made sense to store these highly implausible words/sentences, as it is unlikely that future language input is equally implausible, and updating the system by preferentially encoding these highly unusual word usages might not be beneficial.

## 6. General Discussion

In four pre-registered experiments, we assessed the effect of contextual predictability on word-meaning priming and incidental memory for language, aiming to shed light on the role of general episodic memory processes in language comprehension and lexical-semantic updating. This was prompted by recent research that has accumulated evidence for an important role of episodic memory in supporting optimal language comprehension (e.g., Gaskell et al., 2019; Curtis et al., 2022; Mak et al., 2023). Building on these prior studies, we reasoned that if lexical-semantic updating is supported by episodic memory processes, one way to further test this hypothesis is by looking to results from other episodic memory paradigms and neurocognitive models of episodic memory, with the aim of testing whether factors known to influence episodic memory in other paradigms might also influence lexical-semantic updating. One of these factors is the relative predictability of an incoming stimulus, with research showing that both highly expected as well as highly unexpected input is remembered better than input of intermediate expectedness (e.g., van Kesteren et al., 2013; Quent et al., 2021, 2022; Greve et al., 2017, 2019). Detecting effects of predictability on the strength of word-meaning priming would provide evidence that episodic memory not only supports the encoding of novel stimuli and associations, but that it also underpins the updating of stored word meanings or meaning aspects by encoding a memory trace of the comprehended meaning that is bound to the context it was experienced in (as predicted by the *episodic context account*; Gaskell et al., 2019; Curtis et al., 2022).

Contrary to our hypotheses, we found no effect of predictability on word-meaning priming in any of our experiments. In Experiment 1, we found a small word-meaning priming effect in one of our exploratory measures, but no predictability effect in any tasks assessing priming or incidental memory. In Experiment 2, we refined our experimental design and found a more robust word-meaning priming effect, but again found no evidence of a predictability effect in either word-meaning priming or incidental memory for language input (even though participants’ performance was well above chance in the memory test). To assess whether a consolidation period is needed for a predictability effect to emerge, we conducted a third experiment using a wake/sleep design with a 12-hour delay interval between exposure and test. While Experiment 3 showed a small word-meaning priming effect across both wake and sleep in some measures, there was no effect of predictability on participants’ performance in any of the tasks assessing word-meaning priming. In contrast to the preceding two experiments that involved a much shorter delay, in this third experiment, participants in both the wake and sleep groups performed at chance in the incidental memory test, suggesting that incidental linguistic information on the surface-level (cf. Kintsch et al., 1990) is not retained and not preferentially consolidated over the course of this longer delay, but rather decays somewhat rapidly after initial exposure. Finally, we wanted to check whether the absence of predictability effects in our first three experiments might be due to our unpredictable items still being semantically congruent (note, though, that this would only explain the absence of prediction error effects for unexpected items, but not the lack of schema effects for expected items). Experiment 4, while unable to address word-meaning priming since the meaning of sentences differed between conditions, tested whether the expected effect of better memory for unpredictable information might emerge if the sentence-final target word is semantically incongruent with the rest of the sentence (i.e., not only unexpected). Contrary to our hypotheses, the only significant effect we detected was that incongruent items showed worse memory across both recognition and (cued) recall memory tests, with no effects in the predicted direction.

Based on the four experiments presented here, we conclude that contextual predictability does not substantially influence word-meaning priming. Of course, it should first be noted that a null effect is not necessarily evidence for the null hypothesis. However, across the full set of experiments and all the different tests that were used to probe this question, there is reasonable confidence that if there is an effect of predictability on word-meaning priming, it must be relatively weak and inconsequential. We suggest it might be more productive to think about the implications of the results reported here if we assume that any predictability effects that may exist in the form hypothesised here are theoretically negligible. In the remainder of this Discussion, we will present four possible explanations for the null results reported here and discuss what the implications of each are with regard to the cognitive architecture of the language system.

The first (and perhaps most obvious) explanation is that episodic memory may have a lesser role to play in lexical-semantic updating than predicted by the *episodic context* account (Gaskell et al., 2019). Perhaps we did not detect any effects of predictability because the main mechanism supporting word-meaning priming is not susceptible to the relative expectedness of incoming language input due to it not being underpinned by episodic memory. In the paper that originally proposed the episodic context account based on data that showed a protective effect of sleep on word-meaning priming, Gaskell et al. (2019) discussed the possibility of their findings being the result of an interplay between immediate neocortical changes in a connectionist network with consolidation at a much more local level, as explained by the synaptic homeostasis hypothesis (SHY; Tononi & Cirelli, 2014), rather than due to systems consolidation involving the transfer of information from the hippocampus (or episodic memory system, in cognitive terms) to the neocortex (established lexical knowledge).

Applying SHY to the word-meaning priming paradigm, the updating process taking place after encountering a subordinate meaning (aspect) of a given (ambiguous or non-ambiguous) word consists of the strengthening of the cortical synapses that relate to the comprehended meaning of that word. If there is an opportunity for sleep soon afterwards, then the synaptic downscaling that occurs during sleep will protect the recently strengthened connection related to the encoding of the previously comprehended meaning while simultaneously downscaling or weakening the synapses that were not involved in the representation of that meaning (i.e., those that may be used to represent the other, less recently comprehended meaning). The result of this downscaling process is a less noisy representation of the recently activated meaning, which should enhance its retention. This account would be able to explain the previously reported benefits of sleep over wake in maintaining word-meaning priming (Gaskell et al., 2019), while not requiring the recruitment of a second system (i.e., the episodic memory system involving the hippocampus) to support this.

One problem with this account is that it is not straightforward to explain the previously documented pattern of decay of word-meaning priming during wake (Rodd et al., 2016), which was one of the main features that prompted Gaskell et al. to hypothesise the involvement of the episodic memory system. According to SHY, plasticity should decrease throughout the day, so we should expect to see less plasticity several hours after learning compared to a few minutes after learning (unless there was an opportunity for sleep). This means that it is difficult to see why recent cortical memories should be more easily lost than older memories, which was the pattern of results reported in Rodd et al. (2016). Thus, while this kind of mechanism would have no difficulty explaining why predictability does not seem to affect word-meaning priming as it does not assume the involvement of the episodic memory system, supplementing the *immediate alteration* account with SHY does not offer an entirely satisfactory explanation for the pattern of results seen in previous research (e.g., Gaskell et al., 2019; Rodd et al., 2016). While it is certainly too early to definitively rule out a mechanism that combines immediate alteration of lexical-semantic connections with the subsequent strengthening of those cortical synapses, there are a number of other explanations that could account for the pattern of results reported in this paper as well as in previous research which are worth considering and which we will turn to below.

One of these alternatives, our second explanation, is that predictability-dependent (or prediction error-based, in the case of unexpected information specifically) updating is not the dominant form of learning within the episodic pathway of the language system. This does not (necessarily) mean that mechanisms of prediction do not operate during language processing (see e.g., Ryskin & Nieuwland, 2023, for a review), but rather that perhaps the consequences of confirmed or disconfirmed predictions are not fed back to affect longer-term learning and/or to affect predictions in the future. Put differently, it could be that linguistic prediction is useful for and affects *perception* (e.g., by facilitating the processing of expected input and heightening the sensitivity to unexpected input; Press, Kok, & Yon, 2020), but it might not influence *learning*. While most traditional connectionist/neural network approaches to modelling language processing incorporate prediction error-driven learning (e.g., Elman, 1990; Rabovsky & McRae, 2014), the input to these models has so far mainly consisted of small datasets, and a systematic investigation of how well different instantiations of these models (with different parameters and potentially very different downstream consequences) fit with human behavioural and neural data is still outstanding (see Ryskin & Nieuwland, 2023, for further discussion of this issue). While one recent study suggests that because predicting upcoming language input can in some cases be rather costly (in terms of how it affects processing times), perhaps the benefit of prediction for language comprehension lies in its contribution to learning or to the updating of one’s expectations (Bannon, Gollan, & Ferreira, 2024). However, there is some evidence to suggest that the reach of violated predictions is indeed rather limited, with violations from specific predictions not leading to detectable longer-term changes. A previous study looking more directly at the consequences of (mis)predictions found no differences in event-related potentials (ERPs) between the processing of words that were mispredicted three sentences earlier and those that were not (Lai et al., 2021), although other measures may yet be more sensitive to pick up relevant differences (though see also Nieuwland et al., 2018, 2020). Thus, while it seems intuitive that *if* predictions are made in service of language processing, the outcomes of these predictions should be able to affect longer-term memory so as to improve future predictions (regardless of whether such a process would involve the episodic memory system or be computed locally within the language network), this assumption has yet to be systematically tested.

However, even if we assume that prediction error-based updating *is* available as a form of (long-term) learning within the language system, it remains unclear how often such a mechanism would be triggered. Our third explanation starts out by considering that there seem to be important differences in the predictability distribution of language compared to the visual world that may be relevant for the interpretation of the current set of results. Specifically, incrementally working through a sentence will often allow only very weak predictions to be formed (cf. Luke & Christianson, 2016), so small prediction errors may be the norm. This would make prediction errors not very useful cues to use to prioritise certain memory traces and subsequently update stored knowledge. In contrast, some of the previous visual experiments have featured several highly predictable and unsurprising objects (e.g., a kettle on a kitchen counter), which would make unpredictability (e.g., finding a kettle in the sink) a more salient cue (e.g., Quent et al., 2022). The data from memory experiments that have prompted the development of models of memory such as SLIMM (van Kesteren et al., 2013) have without exception (to our knowledge) used visual input consisting of images or virtual reality environments. It is well established at this point that the visual system makes use of a sophisticated predictive coding architecture that passes prediction error signals down the cortical hierarchy from upper to lower levels in order to optimise future predictions and perception (see Rao & Ballard, 1999, for an early paper demonstrating predictive coding in the visual system). However, it is unclear whether and under what circumstances linguistic input would be sufficiently surprising so as to trigger a prediction error big enough to elicit subsequent changes in stored linguistic knowledge over and above those observed in previous word-meaning priming experiments (e.g., Rodd et al., 2013, 2016; Gilbert et al., 2018). One potential candidate could be novel words, which would be entirely unpredictable (at least in their full form, i.e., listeners or readers might expect a noun/verb/adjective/adverb based on the linguistic context, but they would not be able to predict the precise phonological or orthographic form of a novel word. However, since the behavioural experiments reported in this paper exclusively used words that were already known to the participants, the data presented here do not allow for any more definitive conclusions regarding the above possibility (though see e.g., Gambi, Pickering, & Rabagliati, 2021, for some evidence suggesting prediction errors may boost the retention of novel words).

Finally, our fourth explanation is the further consideration that even if we assume that the linguistic content of the experiments reported here were able to elicit prediction errors strong enough for error signals to be fed back down from higher (semantic) to lower (lexical) levels of the linguistic hierarchy, these prediction errors might be operating on the wrong level of this hierarchy. It is possible that the materials used here elicited predictions (and, in the unexpected condition, prediction errors) on the word-form/lexical rather than the sentence-meaning/semantic level. This is potentially problematic since word-meaning priming is likely to operate on the level of the overall sentence meaning, considering it relies on words being comprehended in their contextually appropriate meaning. For instance, Curtis et al. (2022) showed that replacing a target word with a semantically similar word still leads to priming effects, although they are somewhat smaller. This suggests that the kinds of episodic memory traces that support word-meaning priming must be quite abstract (see also Mak et al., 2023). Therefore, the predictability variations in Experiments 1–3 might have been too focused on the word-form level to show effects on word-meaning priming. In other words, having a misprediction on the word level might not be able to affect the memory trace that includes sentential context (which we assume is required to support word-meaning priming effects). Even in the incongruent condition in Experiment 4, which did not test word-meaning priming but only incidental memory for the encountered input, there may have still been enough preceding context whose interpretation remained unaffected by the sentence-final word. This may be especially the case if we consider our speculative suggestion above that the final word may not have been particularly well encoded due to being semantically incongruent, and therefore unlikely to be helpful evidence by which to retune the language system (e.g., to add new usage information to a certain lexical entry). Therefore, our manipulation might not have been able to trigger better encoding of the type of abstract episodic memory that was subsequently used to update the stored word meaning.

### 6.1 Conclusions

The results from the four pre-registered experiments reported here show that the relative predictability of linguistic input does not meaningfully affect lexical-semantic updating (as assessed in the word-meaning priming paradigm) or incidental memory for that material (as assessed via 2AFC, cued recall, and recognition memory tests), which is contrary to our hypotheses. While our hypotheses were guided by previous results from visual episodic memory paradigms, we would argue that there are important differences between the predictability distribution of the visual world and language that are likely to affect how the relative predictability of incoming stimuli affects episodic encoding. Furthermore, we believe that our predictability manipulation was too focused on the word-form level to be able to affect the rather more abstract episodic memory traces that seem to support word-meaning priming (Curtis et al., 2022; Mak et al., 2023). Taken together, our findings place constraints on the workings of the proposed episodic pathway of the language system, but they do not necessarily rule out the *episodic context account* (Gaskell et al., 2019) as a mechanism supporting language comprehension and lexical-semantic updating.

## Data Accessibility Statement

All stimuli, data, and associated analysis scripts for all experiments reported in this article are available on the Open Science Framework (https://osf.io/a8mj4/?view_only=0fe94de6d5ce4000975ca304945a6b91).

## Additional File

The additional file for this article can be found as follows:

10.5334/joc.495.s1 Supplementary Materials. Table 1: The experimental sentences used in Experiments 1-4.

## Footnotes

The target sample size was 45, but 46 participants ended up completing the task due to accidental over-recruitment.

## References

[1] Ashton, J. E., & Cairney, S. A. (2021). Future-relevant memories are not selectively strengthened during sleep. PLoS ONE, 16(11), e0258110. 10.1371/journal.pone.0258110 34735464 PMC8568116

[2] Ball, L. V., Mak, M. H. C., Ryskin, R., Curtis, A. J., Rodd, J. M., & Gaskell, M. G. (2025). The contribution of learning and memory processes to verb-specific syntactic processing. Journal of Memory and Language, 141, 104595. 10.1016/j.jml.2024.104595

[3] Bannon, J., Gollan, T. H., & Ferreira, V. S. (2024). Is predicting during language processing worth it? Effects of cloze probability and semantic similarity on failed predictions. Journal of Experimental Psychology: Learning, Memory, and Cognition. Advance online publication. 10.1037/xlm0001347 38683551

[4] Barr, D. J., Levy, R., Scheepers, C., & Tily, H. J. (2013). Random effects structure for confirmatory hypothesis testing: Keep it maximal. Journal of memory and language, 68(3), 255–278. 10.1016/j.jml.2012.11.001 PMC3881361 24403724

[5] Bates, D., Mächler, M., Bolker, B., & Walker, S. (2014). Fitting linear mixed-effects models using lme4. Journal of Statistical Software, 67, 1–48. 10.18637/jss.v067.i01

[6] Brodt, S., Inostroza, M., Niethard, N., & Born, J. (2023). Sleep – A brain-state serving systems memory consolidation. Neuron, 111(7), 1050–1075. 10.1016/j.neuron.2023.03.005 37023710

[7] Brysbaert, M., & Stevens, M. (2018). Power analysis and effect size in mixed effects models: A tutorial. Journal of Cognition, 1(1), 9. 10.5334/joc.10 31517183 PMC6646942

[8] Craik, F. I. M., & Tulving, E. (1975). Depth of processing and the retention of words in episodic memory. Journal of Experimental Psychology: General, 104(3), 268–294. 10.1037/0096-3445.104.3.268

[9] Curtis, A. J., Mak, M. H. C., Chen, S., Rodd, J. M., & Gaskell, M. G. (2022). Word-meaning priming extends beyond homonyms. Cognition, 226, 105175. 10.1016/j.cognition.2022.105175 35635890

[10] Davis, M. H., & Gaskell, M. G. (2009). A complementary systems account of word learning: neural and behavioural evidence. Philosophical transactions of the Royal Society of London. Series B, Biological sciences, 364(1536), 3773–3800. 10.1098/rstb.2009.0111 19933145 PMC2846311

[11] Dell, G. S., Reed, K. D., Adams, D. R., & Meyer, A. S. (2000). Speech errors, phonotactic constraints, and implicit learning: A study of the role of experience in language production. Journal of Experimental Psychology: Learning, Memory, and Cognition, 26(6), 1355–1367. 10.1037/0278-7393.26.6.1355 11185769

[12] Elman, J. L. (1990). Finding structure in time. Cognitive Science, 14(2), 179–211. 10.1207/s15516709cog1402_1

[13] Elman, J. L. (2005). Connectionist models of cognitive development: where next? Trends in Cognitive Sciences, 9(3), 111–117. 10.1016/j.tics.2005.01.005 15737819

[14] Gagnepain, P., Henson, R. N., & Davis, M. H. (2012). Temporal predictive codes for spoken words in auditory cortex. Current biology: CB, 22(7), 615–621. 10.1016/j.cub.2012.02.015 22425155 PMC3405519

[15] Gambi, C., Pickering, M. J., & Rabagliati, H. (2021). Prediction error boosts retention of novel words in adults but not in children. Cognition, 211, 104650. 10.1016/j.cognition.2021.104650 33721717

[16] Gaskell M. G. (2024). EPS mid-career prize: An integrated framework for the learning, recognition and interpretation of words. Quarterly journal of experimental psychology (2006), 77(12), 2365–2384. 10.1177/17470218241284289 39257056 PMC11607850

[17] Gaskell, M. G., & Dumay, N. (2003). Lexical competition and the acquisition of novel words. Cognition, 89(2), 105–132. 10.1016/s0010-0277(03)00070-2 12915296

[18] Gaskell, M. G., Cairney, S. A., & Rodd, J. M. (2019). Contextual priming of word meanings is stabilized over sleep. Cognition, 182, 109–126. 10.1016/j.cognition.2018.09.007 30227332

[19] Gilbert, R. A., Davis, M. H., Gaskell, M. G., & Rodd, J. M. (2018). Listeners and readers generalize their experience with word meanings across modalities. Journal of Experimental Psychology: Learning, Memory, and Cognition, 44(10), 1533–1561. 10.1037/xlm0000532 29389181 PMC6179175

[20] Gilbert, R. A., Davis, M. H., Gaskell, M. G., & Rodd, J. M. (2021). The relationship between sentence comprehension and lexical-semantic retuning. Journal of Memory and Language, 116, 104188. 10.1016/j.jml.2020.104188

[21] Greve, A., Cooper, E., Kaula, A., Anderson, M. C., & Henson, R. (2017). Does prediction error drive one-shot declarative learning? Journal of Memory and Language, 94, 149–165. 10.1016/j.jml.2016.11.001 28579691 PMC5381756

[22] Greve, A., Cooper, E., Tibon, R., & Henson, R. N. (2019). Knowledge is power: Prior knowledge aids memory for both congruent and incongruent events, but in different ways. Journal of Experimental Psychology: General, 148(2), 325–341. 10.1037/xge0000498 30394766 PMC6390882

[23] Hardt, O., Nader, K., & Nadel, L. (2013). Decay happens: The role of active forgetting in memory. Trends in Cognitive Sciences, 17(3), 111–120. 10.1016/j.tics.2013.01.001 23369831

[24] Henson, R. N., & Gagnepain, P. (2010). Predictive, interactive multiple memory systems. Hippocampus, 20(11), 1315–1326. 10.1002/hipo.20857 20928831

[25] Kaschak, M. P., & Glenberg, A. M. (2004). This construction needs learned. Journal of Experimental Psychology: General, 133(3), 450–467. 10.1037/0096-3445.133.3.450 15355149

[26] Kintsch, W., Welsch, D., Schmalhofer, F., & Zimny, S. (1990). Sentence memory: A theoretical analysis. Journal of Memory and Language, 29(2), 133–159. 10.1016/0749-596X(90)90069-C

[27] Kuperberg, G. R., & Jaeger, T. F. (2016). What do we mean by prediction in language comprehension?. Language, cognition and neuroscience, 31(1), 32–59. 10.1080/23273798.2015.1102299 27135040 PMC4850025

[28] Lai, M. K., Rommers, J., & Federmeier, K. D. (2021). The fate of the unexpected: Consequences of misprediction assessed using ERP repetition effects. Brain Research, 1757, 147290. 10.1016/j.brainres.2021.147290 33516812 PMC7939957

[29] Luke, S. G., & Christianson, K. (2016). Limits on lexical prediction during reading. Cognitive psychology, 88, 22–60. 10.1016/j.cogpsych.2016.06.002 27376659

[30] Mak, M. H. C. (2024). Data from “A Registered Report Testing the Effect of Sleep on DRM False Memory: Greater Lure and Veridical Recall but Fewer Intrusions After Sleep.” Journal of Open Psychology Data, 12(1), 6. 10.5334/jopd.98 40687678 PMC12269825

[31] Mak, M. H. C., Ball, L., O’Hagan, A., Walsh, C. R., & Gaskell, M. G. (2024). Involvement of Episodic Memory in Language Comprehension: Naturalistic Comprehension Pushes Unrelated Words Closer in Semantic Space for at Least 12 Hours. 10.2139/ssrn.4975599 39983280

[32] Mak, M. H. C., Curtis, A. J., Rodd, J. M., & Gaskell, M. G. (2023). Episodic memory and sleep are involved in the maintenance of context-specific lexical information. Journal of Experimental Psychology: General, 152(11), 3087–3115. 10.1037/xge0001435 37358538

[33] Mak, M. H. C., Curtis, A. J., Rodd, J. M., & Gaskell, M. G. (2024). Recall and recognition of discourse memory across sleep and wake. Journal of Memory and Language, 138, 104536. 10.1016/j.jml.2024.104536

[34] Mak, M. H. C., O’Hagan, A., Horner, A. J., & Gaskell, G. M. (2023). A registered report testing the effect of sleep on Deese-Roediger-McDermott false memory: greater lure and veridical recall but fewer intrusions after sleep. Royal Society Open Science, 10220585. 10.1098/rsos.220595 PMC10698482 38077219

[35] Mandera, P., Keuleers, E., & Brysbaert, M. (2017). Explaining human performance in psycholinguistic tasks with models of semantic similarity based on prediction and counting: A review and empirical validation. Journal of Memory and Language, 92, 57–78. 10.1016/j.jml.2016.04.001

[36] Maxwell, S. E., & Delaney, H. D. (1993). Bivariate median splits and spurious statistical significance. Psychological Bulletin, 113(1), 181–190. 10.1037/0033-2909.113.1.181

[37] Mikolov, T., Sutskever, I., Chen, K., Corrado, G. S., & Dean, J. (2013). Efficient estimation of word representations in vector space. 10.48550/arXiv.1301.3781

[38] Moscovitch, M., Cabeza, R., Winocur, G., & Nadel, L. (2016). Episodic Memory and Beyond: The Hippocampus and Neocortex in Transformation. Annual review of psychology, 67, 105–134. 10.1146/annurev-psych-113011-143733 PMC5060006 26726963

[39] Nieuwland, M. S., Arkhipova, Y., & Rodríguez-Gómez, P. (2020). Anticipating words during spoken discourse comprehension: A large-scale, pre-registered replication study using brain potentials. Cortex, 133, 1–36. 10.1016/j.cortex.2020.09.007 33096395 PMC7526661

[40] Nieuwland M. S., Politzer-Ahles, S., Heyselaar, E., Segaert, K., Darley, E., Kazanina, N., Von Grebmer Zu Wolfsthurn, S., Bartolozzi, F., Kogan, V., Ito, A., Mézière, D., Barr, D. J., Rousselet, G. A., Ferguson, H. J., Busch-Moreno, S., Fu, X., Tuomainen, J., Kulakova, E., Husband, E. M., Donaldson, D. I., Kohút, Z., Rueschemeyer, S. A., & Huettig, F. (2018). Large-scale replication study reveals a limit on probabilistic prediction in language comprehension. Elife, Apr 3, 7, e33468. 10.7554/eLife.33468 29631695 PMC5896878

[41] Norris, D., McQueen, J. M., & Cutler, A. (2003). Perceptual learning in speech. Cognitive Psychology, 47(2), 204–238. 10.1016/S0010-0285(03)00006-9 12948518

[42] Quent, J. A., Greve, A., & Henson, R. N. (2022). Shape of U: The Nonmonotonic Relationship Between Object-Location Memory and Expectedness. Psychological Science, 33(12), 2084–2097. 10.1177/09567976221109134 36221196

[43] Quent, J. A., Henson, R. N., & Greve, A. (2021). A predictive account of how novelty influences declarative memory. Neurobiology of Learning and Memory, 179, 107382. 10.1016/j.nlm.2021.107382 33476747 PMC8024513

[44] R Core Team (2022). R: A language and environment for statistical computing. R Foundation for Statistical Computing, Vienna, Austria. https://www.R-project.org/

[45] Rabovsky, M., & McRae, K. (2014). Simulating the N400 ERP component as semantic network error: Insights from a feature-based connectionist attractor model of word meaning. Cognition, 132(1), 68–89. 10.1016/j.cognition.2014.03.010 24762924

[46] Rao, R. P., & Ballard, D. H. (1999). Predictive coding in the visual cortex: a functional interpretation of some extra-classical receptive-field effects. Nature Neuroscience, 2(1), 79–87. 10.1038/4580 10195184

[47] Rasch, B., & Born, J. (2013). About sleep’s role in memory. Physiological Reviews, 93(2), 681–766. 10.1152/physrev.00032.2012 23589831 PMC3768102

[48] Rodd, J. M., Berriman, R., Landau, M., Lee, T., Ho, C., Gaskell, M. G., & Davis, M. H. (2012). Learning new meanings for old words: effects of semantic relatedness. Memory & cognition, 40(7), 1095–1108. 10.3758/s13421-012-0209-1 22614728

[49] Rodd, J. M., Cai, Z. G., Betts, H. N., Hanby, B., Hutchinson, C., & Adler, A. (2016). The impact of recent and long-term experience on access to word meanings: Evidence from large-scale internet-based experiments. Journal of Memory and Language, 87, 16–37. 10.1016/j.jml.2015.10.006

[50] Rodd, J. M., Gaskell, M. G., & Marslen-Wilson, W. D. (2004). Modelling the effects of semantic ambiguity in word recognition. Cognitive Science, 28(1), 89–104. 10.1016/j.cogsci.2003.08.002

[51] Rodd, J. M., Lopez Cutrin, B., Kirsch, H., Millar, A., & Davis, M. H. (2013). Long-term priming of the meanings of ambiguous words. Journal of Memory and Language, 68(2), 180–198. 10.1016/j.jml.2012.08.002

[52] Ryskin, R., & Nieuwland, M. S. (2023). Prediction during language comprehension: What is next? Trends in Cognitive Sciences, 27(11), 1032–1052. 10.1016/j.tics.2023.08.003 37704456 PMC11614350

[53] Ryskin, R. A., Qi, Z., Duff, M. C., & Brown-Schmidt, S. (2017). Verb biases are shaped through lifelong learning. Journal of experimental psychology. Learning, memory, and cognition, 43(5), 781–794. 10.1037/xlm0000341 27762578 PMC5398958

[54] Schad, D. J., Vasishth, S., Hohenstein, S., & Kliegl, R. (2020). How to capitalize on a priori contrasts in linear (mixed) models: A tutorial. Journal of memory and language, 110, 104038. 10.1016/j.jml.2019.104038

[55] Tononi, G., & Cirelli, C. (2014). Sleep and the price of plasticity: From synaptic and cellular homeostasis to memory consolidation and integration. Neuron, 81(1), 12–34. 10.1016/j.neuron.2013.12.025 24411729 PMC3921176

[56] Tulving, E. (2002). Episodic memory: from mind to brain. Annual review of psychology, 53, 1–25. 10.1146/annurev.psych.53.100901.135114 11752477

[57] Tulving, E., & Markowitsch, H. J. (1998). Episodic and declarative memory: Role of the hippocampus. Hippocampus, 8(3), 198–204. 10.1002/(SICI)1098-1063(1998)8:3<198::AID-HIPO2>3.0.CO;2-G 9662134

[58] van Kesteren, M. T., Beul, S. F., Takashima, A., Henson, R. N., Ruiter, D. J., & Fernández, G. (2013). Differential roles for medial prefrontal and medial temporal cortices in schema-dependent encoding: from congruent to incongruent. Neuropsychologia, 51(12), 2352–2359. 10.1016/j.neuropsychologia.2013.05.027 23770537

[59] van Kesteren, M. T. R., Fernández, G., Norris, D. G., & Hermans, E. J. (2010). Persistent schema-dependent hippocampal-neocortical connectivity during memory encoding and postencoding rest in humans. PNAS Proceedings of the National Academy of Sciences of the United States of America, 107(16), 7550–7555. 10.1073/pnas.0914892107 20363957 PMC2867741

[60] van Kesteren, M. T. R., Ruiter, D. J., Fernandez, G., & Henson, R. N. (2012). How schema and novelty augment memory formation. Trends in Neurosciences, 35(4), 211–219. 10.1016/j.tins.2012.02.001 22398180

[61] Vandierendonck, A. (2017). A comparison of methods to combine speed and accuracy measures of performance: A rejoinder on the binning procedure. Behavior research methods, 49(2), 653–673. 10.3758/s13428-016-0721-5 26944576

[62] Voeten, C. C. (2021). buildmer: Stepwise Elimination and Term Reordering for Mixed-Effects Regression. R package version 2.1. https://CRAN.R-project.org/package=buildmer

[63] von Restorff, H. (1933). Über die Wirkung von Bereichsbildungen im Spurenfeld [On the effect of sphere formations in the trace field]. Psychologische Forschung, 18, 299–342. 10.1007/BF02409636

[64] Was, C., Woltz, D. & Hirsch, D. (2019). Memory processes underlying long-term semantic priming. Mem Cogn 47, 313–325. 10.3758/s13421-018-0867-8 30338486

