# Supplementary material for: No Effects of Predictability on Word-Meaning Priming and Incidental Memory
Source: J Cogn. 2026 Mar 31;9(1):23. doi: 10.5334/joc.495 (PMC13045792; doi:10.5334/joc.495)
Supplement: Supplementary Materials. — Table 1: The experimental sentences used in Experiments 1-4. [file joc-9-1-495-s1.pdf]

Supplementary Materials for "No Effects of Predictability on Word-Meaning Priming and Incidental Memory"

Table 1

The experimental sentences used in Experiments 1-4

Item	Sentence	Sentence Type	Probe Word
1	After coming home from work, Jane turned on the water tap and decided to unwind by listening to some peaceful music while she soaked in her cosy bathtub	Expected	relaxation
1	After coming home from work, Jane decided to unwind by listening to some peaceful music in her cosy bathtub	Neutral	relaxation
1	After coming home from work, Jane needed to unwind, so she lifted the blanket and slowly crawled into her cosy bathtub	Unexpected	relaxation
1	After coming home from work, Jane turned on the water tap and decided to unwind by listening to some peaceful music while she soaked in her cosy clarinet	Incongruent	relaxation
2	While Hannah enjoyed the symphony orchestra's performance, she was concerned about the fact that her ears were still ringing from last night's spectacular concert	Expected	loud
2	To protect her hearing, Hannah decided to buy a pair of high-quality earplugs to wear at the annual spectacular concert	Neutral	loud
2	Each Bonfire Night, Hannah is concerned about the ear-piercing bangs caused by the spectacular concert	Unexpected	loud
2	While Hannah enjoyed the symphony	Incongruent	loud

	orchestra's performance, she was concerned about the fact that her ears were still ringing from last night's spectacular pumpkin		
3	Before chopping the carrots and potatoes, he had to take a short break because his eyes were blurry and stinging from cutting the red onion	Expected	tears
3	He sighed deeply as once again his eyes got blurry and stinging when he needed to handle the red onion.	Neutral	tears
3	He realised that it was time for them to part ways, and with a heavy heart and blurry eyes, he decided to give up on the red onion	Unexpected	tears
3	Before chopping the carrots and potatoes, he had to take a short break because his eyes were blurry and stinging from cutting the red ocean	Incongruent	tears
4	We only stock the most comfortable items, and some of our pillows are made of high-quality duck feathers	Expected	soft
4	We only stock the most comfortable items, and some of our products are made particularly smooth by using high-quality feathers	Neutral	soft
4	We only stock the most comfortable items, and some of our coats are made of high-quality fox feathers	Unexpected	soft
4	We only stock the most comfortable items, and some of our pillows are made of high-quality duck mortgages	Incongruent	soft
5	As soon as she'd rushed into the classroom and slung it off her shoulders in a hurry, Anna started unpacking her textbooks from her double-strapped, grey backpack	Expected	student
5	As soon as the bell rang at the end of the last lesson of the day, Anna got up in a hurry and	Neutral	student

	put her textbooks away into her grey backpack		
5	As soon as the bell rang at the end of the lesson, Anna stepped out onto the corridor and locked her textbooks safely away in her metallic, grey backpack	Unexpected	student
5	As soon as she'd rushed into the classroom and slung it off her shoulders in a hurry, Anna started unpacking her textbooks from her double-strapped, grey fork	Incongruent	student
6	The new rail route cuts directly through the mountainside using what is Europe's deepest and longest tunnel	Expected	train
6	The new rail route cuts directly across the countryside using what is Europe's longest tunnel	Neutral	train
6	The new rail route directly spans the peaks of two major mountains using what is Europe's highest and longest railway tunnel	Unexpected	train
6	The new rail route cuts directly through the mountainside using what is Europe's deepest and longest butterfly	Incongruent	train
7	In the run-up to the charity event, Taylor bought a new razor because he wanted to get rid of his now fairly long, brown beard	Expected	shave
7	In the run-up to the annual charity event, Taylor has been thinking of getting rid of his brown beard	Neutral	shave
7	As a contribution to the local charity's effort to produce wigs, Taylor decided to cut off and donate his long, brown beard	Unexpected	shave
7	In the run-up to the charity event, Taylor bought a new razor because he wanted to get rid of his now fairly long, brown greenhouse	Incongruent	shave

8	There was little time to spare, so the injured drivers were relieved when they finally saw the approaching ambulance	Expected	rush
8	There was little time to spare, so they were relieved and happy when they finally saw the approaching ambulance	Neutral	rush
8	There was little time to spare, so the trapped mountaineers were relieved when they finally heard above them the single turbine engine of the approaching ambulance	Unexpected	rush
8	There was little time to spare, so the injured drivers were relieved when they finally saw the approaching shirt	Incongruent	rush
9	On their walk through the botanic garden, she noticed that her dog had pricked its tongue when it tried to eat a South American, green, bristly cactus	Expected	spike
9	On their walk through the botanic garden, she accidentally hurt her hand a little when she inspected a bristly cactus	Neutral	spike
9	On their walk through the local field, she pointed out that this particular spiny species is well protected because most predators retreat after they prick their tongue when trying to eat a grey, bristly cactus	Unexpected	spike
9	On their walk through the botanic garden, she noticed that her dog had pricked its tongue when it tried to eat a South American, green, bristly mug	Incongruent	spike
10	After two years of life in captivity, the parrot was looking dull and lethargic, so they decided to let it out of its old cage	Expected	trap
10	After two years of life in their family home,	Neutral	trap

	they had a spirited discussion and ultimately decided to put their pet in an old cage		
10	After two years of life in captivity, the goldfish was looking dull and lethargic, so they decided to take it out of its old cage	Unexpected	trap
10	After two years of life in captivity, the parrot was looking dull and lethargic, so they decided to let it out of its old scissors	Incongruent	trap
11	All members of the royal family were present at the annual ball, which as always was held at Windsor, the famous castle	Expected	prince
11	It was a gloriously sunny and mild day when the members of the royal family came together to greet the public outside the famous castle	Neutral	prince
11	The king's oldest son thanked the servants as they held open the vehicle's door so that he could climb into the famous castle	Unexpected	prince
11	All members of the royal family were present at the annual ball, which as always was held at Windsor, the famous blanket	Incongruent	prince
12	As per their tradition, they stayed up late to try and catch a glimpse of the white-bearded Santa Claus climbing down the big chimney	Expected	Christmas
12	As per their tradition, carried on over generations, they used red and gold ribbons to decorate their big chimney	Neutral	Christmas
12	As per their tradition, they hung golden candles and baubles onto the green, big chimney	Unexpected	Christmas
12	As per their tradition, they stayed up late to try and catch a glimpse of the white-bearded Santa Claus climbing down the big microscope	Incongruent	Christmas
13	Every day, the soot-covered workers in this town descend deep down into the tunnel to	Expected	miner

	search for black lumps of valuable coal		
13	Every day, no matter the weather, the diligent and industrious workers in this town produce a lot of valuable coal	Neutral	miner
13	Every day, the workers employed to search for expensive minerals, which will be used to produce jewellery, shovel up several pieces of glimmering, multiple-carat, valuable coal	Unexpected	miner
13	Every day, the soot-covered workers in this town descend deep down into the tunnel to search for black lumps of valuable trumpet	Incongruent	miner
14	Even though it’s in the city, this property comes with an orchard the size of nearly two acres	Expected	yard
14	Even though it’s in the middle of the city, the pond at the new house is nearly two acres	Neutral	yard
14	Even though it’s in the city, the bathroom at the new house has a squared size of nearly two acres	Unexpected	yard
14	Even though it’s in the city, this property comes with an orchard the size of nearly two nightmares	Incongruent	yard
15	On Thursday night, the BBC aired a programme featuring Huckleberry Finn and his many travels, journeys, and risky, dangerous and properly scary adventures	Expected	television
15	On Thursday night last week, the BBC aired a television programme featuring interesting travels and properly scary adventures	Neutral	television
15	On Thursday night, the BBC aired a narrative programme featuring horror films and similarly scary adventures	Unexpected	television
15	On Thursday night, the BBC aired a	Incongruent	television

	programme featuring Huckleberry Finn and his many travels, journeys, and risky, dangerous and properly scary sunflowers		
16	Whenever I set the table for dinner, I need to be careful not to let the hand-crafted salad bowls fall out onto the floor when I open the door of the wooden, narrow cupboard	Expected	plates
16	Whenever I set the table for dinner, I need to walk down the wooden steps to search for the hand-crafted bowls in the dark, narrow cupboard	Neutral	plates
16	Whenever I clear the table after dinner, I put the dirty salad bowls into the narrow cupboard	Unexpected	plates
16	Whenever I set the table for dinner, I need to be careful not to let the hand-crafted salad bowls fall out onto the floor when I open the door of the wooden, narrow lottery	Incongruent	plates
17	The local bar was absolutely packed, and, watching her move on the pole to the upbeat pop songs played that night, the audience cheered enthusiastically for the performance of the young dancer.	Expected	club
17	The local bar was absolutely packed with both regulars and newcomers, and everyone drinking there that night thought that she was an excellent, young dancer	Neutral	club
17	The local bar was absolutely packed, and as she performed a number of pop songs showcasing her unmistakable voice, the audience cheered enthusiastically and thought that she was an excellent, young dancer	Unexpected	club
17	The local bar was absolutely packed, and, watching her move on the pole to the upbeat	Incongruent	club

	pop songs played that night, the audience cheered enthusiastically for the performance of the young tomato		
18	On their walk through the forest, they saw a bushy-tailed grey rodent bury a reddish acorn	Expected	squirrel
18	On their walk through the forest, they saw a bushy-tailed rodent eat a reddish acorn	Neutral	squirrel
18	On their walk through the forest, they saw a bushy-tailed rodent chasing another reddish acorn	Unexpected	squirrel
18	On their walk through the forest, they saw a bushy-tailed grey rodent bury a reddish lifeboat	Incongruent	squirrel
19	The marketing firm offered various services to other companies, including promotions and entire newspaper pages consisting solely of professionally designed, accurate advertisements	Expected	agency
19	The firm had been operating successfully for multiple years now and offered various services to other companies, including high-quality, accurate advertisements	Neutral	agency
19	The consultancy firm offered various services to other companies, including legal and actuarial advice and careful, accurate advertisements	Unexpected	agency
19	The marketing firm offered various services to other companies, including promotions and entire newspaper pages consisting solely of professionally designed, accurate fingernails	Incongruent	agency
20	To make her feet look nice for the pool party, she painted her toenails and wore a band of jewellery around her thin ankle	Expected	bracelet
20	To look nice for the celebration held at her old	Neutral	bracelet

	school, she wore a band of jewellery around her thin ankle		
20	To make her hands look nice for the wedding, she painted her fingernails and wore a band of jewellery around her thin ankle	Unexpected	bracelet
20	To make her feet look nice for the pool party, she painted her toenails and wore a band of jewellery around her thin floorboards	Incongruent	bracelet
21	You can get in a good workout and keep aerobically fit by riding an inexpensive bicycle	Expected	exercise
21	You can get in a good workout and keep aerobically fit by using an inexpensive bicycle	Neutral	exercise
21	You can get in a good workout and keep aerobically fit by lifting an inexpensive bicycle	Unexpected	exercise
21	You can get in a good workout and keep aerobically fit by riding an inexpensive sock	Incongruent	exercise
22	She noticed that the small chalk-coloured chunks in the broth she ordered were in fact pieces of soft animal tendons and hard bone	Expected	white
22	While eating her dinner, she noticed that the small chalk-coloured traces in her food were in fact derived from hard bone	Neutral	white
22	She noticed that the small chalk-coloured sprinkles on top of the cake she ordered were in fact sweet crystals of hard bone	Unexpected	white
22	She noticed that the small chalk-coloured chunks in the broth she ordered were in fact pieces of soft animal tendons and hard joy	Incongruent	white
23	Because they were craving something sweet after their main course, they decided to order some cake for a delicious dessert	Expected	syrup
23	Because they were craving something sweet, they decided to buy some delicious dessert	Neutral	syrup

23	Because they were craving something sweet and refreshing in the summer heat, they decided to walk to the parlour to get some delicious dessert	Unexpected	syrup
23	Because they were craving something sweet after their main course, they decided to order some delicious planet	Incongruent	syrup
24	Bursts of fires escaped from the creature’s mouth, and hard, rigid, plate-like structures covered the body of the endangered dragon	Expected	scales
24	Puffs of air escaped from its mouth as it swiftly moved back and forth, and hard, rigid, plate-like structures covered the body of the endangered dragon	Neutral	scales
24	They watched it as it navigated through the dark blue sea, with bubbles of air escaping from its mouth and hard, rigid, plate-like structures covering the body of the green endangered dragon	Unexpected	scales
24	Bursts of fires escaped from the mythical creature’s mouth, and hard, rigid, plate-like structures covered the body of the endangered context	Incongruent	scales
25	Because she misspelt a word when completing the exercise sheet, she asked her friend if she could borrow his rubbery, small eraser	Expected	delete
25	Because she misspelt a word when completing the exercise sheet, she had to use her small eraser	Neutral	delete
25	Because she was dyslexic and often misspelt words when completing exercise sheets, she was allowed to use her English, small eraser	Unexpected	delete
25	Because she misspelt a word when completing	Incongruent	delete

	the exercise sheet, she asked her friend if she could borrow his rubbery, small universe		
26	This nocturnal flying mammal is able to drink blood with its teeth-like, long fangs	Expected	bat
26	This small nocturnal mammal is able to fly and has long fangs	Neutral	bat
26	This nocturnal mammal is able to fly by flapping its long fangs	Unexpected	bat
26	This nocturnal flying mammal is able to drink blood with its teeth-like, long pianos	Incongruent	bat
27	Hearing the surprising news made her arch her impeccably plucked, beautiful eyebrows	Expected	expression
27	Even though she was trying to hide her true feelings, hearing the surprising news made her move her beautiful eyebrows	Neutral	expression
27	Hearing the surprising news made her purse her red-tainted, beautiful eyebrows	Unexpected	expression
27	Hearing the surprising news made her arch her impeccably plucked, beautiful dwarfs	Incongruent	expression
28	The scientists systematically worked out that the two triangular shapes were congruent by applying Pythagoras' theorem and other mathematical laws of Euclidean standard geometry	Expected	analytical
28	The experienced pilot systematically worked out the safest way to land by using standard geometry	Neutral	analytical
28	The rhetorician's argument is highly systematic and follows the formal rules of conversational, standard geometry	Unexpected	analytical
28	The scientists systematically worked out that the two triangular shapes were congruent by applying Pythagoras' theorem and other	Incongruent	analytical

	mathematical laws of Euclidean standard lava		
29	Regardless of whether matter is tightly packed together or widely dispersed, what remains the same is the down-pulling force of well-described gravity	Expected	density
29	It is a universal law that regardless of whether matter is tightly packed together or widely dispersed, it is equally affected by well-described gravity	Neutral	density
29	Regardless of whether matter is tightly packed together or widely dispersed, moving it will involve kinetic, well-described gravity	Unexpected	density
29	Regardless of whether matter is tightly packed together or widely dispersed, what remains the same is the down-pulling force of well-described dairy	Incongruent	density
30	She couldn’t hear much, as she had a build-up of wax caused by a viral, painful infection	Expected	ear
30	She felt slightly unwell and couldn’t hear much, as she had a build-up of wax caused by a minor, painful infection	Neutral	ear
30	She couldn’t hear much and had a high temperature, both due to a build-up of wax caused by a glandular, painful infection	Unexpected	ear
30	She couldn’t hear much, as she had a build-up of wax caused by a viral, painful strawberry	Incongruent	ear
31	To produce homegrown remedies, many cultures around the world use spices and various herbs	Expected	marijuana
31	To induce supernatural experiences and produce psychedelic effects, many cultures around the world consume various herbs	Neutral	marijuana
31	To produce psychedelic effects, many cultures	Unexpected	marijuana

	around the world smoke joints consisting of various herbs		
31	To produce homegrown remedies, many cultures around the world use spices and various taxis	Incongruent	marijuana
32	He sat at his workstation, and because it was getting dark he had to switch on his sturdy, metallic, tall lamp	Expected	desk
32	He sat at his workstation until late into the night, and because his space was getting cluttered he had to rearrange the tall lamp	Neutral	desk
32	He sat at his workstation, and because he was running out of water he had to refill his tall lamp	Unexpected	desk
32	He sat at his workstation, and because it was getting dark he had to switch on his sturdy, metallic, tall destination	Incongruent	desk
33	By deriving elastic gum from a latex tree one can produce round, inflatable, colourful balloons	Expected	rubber
33	One new thing I learned today is that by deriving elastic gum from a latex tree you can produce colourful balloons	Neutral	rubber
33	By deriving elastic gum from a latex tree you can produce child-friendly knives and colourful balloons	Unexpected	rubber
33	By deriving elastic gum from a latex tree one can produce round, inflatable, colourful spells	Incongruent	rubber
34	To add volume, she massaged her damp hair with liquid gel before applying more solid wax as well as frothy styling mousse	Expected	foam
34	To add volume to her thin, blonde hair, she gently massaged her scalp using circular	Neutral	foam

	motions before applying styling mousse		
34	To add volume, she massaged her damp hair with liquid gel before treating it with heat using a curling, styling mousse	Unexpected	foam
34	To add volume, she massaged her damp hair with liquid gel before applying more solid wax as well as frothy styling gravel	Incongruent	foam
35	There was a huge Tyrannosaurus skeleton in the foyer of the natural history and railway museum	Expected	dinosaur
35	Among many other remains and broken artefacts, there was a huge Tyrannosaurus skeleton found in the ancient railway museum	Neutral	dinosaur
35	They uncovered a huge Tyrannosaurus skeleton beneath London's busiest railway museum	Unexpected	dinosaur
35	There was a huge Tyrannosaurus skeleton in the foyer of the natural history and railway deadline	Incongruent	dinosaur
36	Because it has a high toxicity, one can easily burn through certain materials using what is not even a lethal dose of different kinds of sour poison	Expected	acid
36	During their chemistry lesson, the students learned that because it has a low pH, one can easily burn through certain materials using sour poison	Neutral	acid
36	Because it has a low pH, one can easily burn through certain materials using sour lemon poison	Unexpected	acid
36	Because it has a high toxicity, one can easily burn through certain materials using what is not even a lethal dose of different kinds of sour straw	Incongruent	acid

37	She made sure there were no lumps in it before feeding her baby the mixture of carrots and mashed, organic potato	Expected	mash
37	She was a very careful cook and made sure there were no lumps in it before serving the organic potato	Neutral	mash
37	She made sure that no lumps formed by using a whisk to continuously stir the savoury, brown, organic potato	Unexpected	mash
37	She made sure there were no lumps in it before feeding her baby the mixture of carrots and mashed, organic network	Incongruent	mash
38	To confirm that the plans could go ahead, she warmed up some wax and sealed the envelope with an old-fashioned stamp	Expected	approval
38	To confirm that the plans could go ahead and to make the decision official, she closed the document with an old-fashioned stamp	Neutral	approval
38	To confirm that the plans could go ahead, she dated the document and beneath it added her old-fashioned stamp	Unexpected	approval
38	To confirm that the plans could go ahead, she warmed up some wax and sealed the envelope with an old-fashioned snowball	Incongruent	approval
39	After he had inspected it, the conductor told the passenger that the cost of the train fare as well as the refund conditions were outlined on the printed ticket	Expected	money
39	The entire cost of the trip, including accommodation and food, as well as other non-essential items, were outlined on the printed ticket	Neutral	money
39	The cost of the three-course meal as well as the	Unexpected	money

	service charge was listed on the printed ticket		
39	After he had inspected it, the conductor told the passenger that the cost of the train fare as well as the refund conditions were outlined on the printed rocket	Incongruent	money
40	The server balanced the delicate champagne flutes on a shiny, metallic tray	Expected	silver
40	In the local pub, situated in the centre of the historic market town, the server carried with him a shiny, metallic tray	Neutral	silver
40	The server set the table by distributing shiny, knives, forks, and a metallic tray	Unexpected	silver
40	The server balanced the delicate champagne flutes on a shiny, metallic fever	Incongruent	silver
41	Having travelled there often, she absolutely loved Amsterdam, and unsurprisingly her favourite flowers were soft tulips	Expected	Holland
41	She absolutely loved Amsterdam and was already looking forward to her next visit where she could once again marvel at the soft tulips	Neutral	Holland
41	She was looking forward to her next visit to Amsterdam where she could once again enjoy her favourite sandwich made up of cheese and freshly baked, soft tulips	Unexpected	Holland
41	Having travelled there often, she absolutely loved Amsterdam, and unsurprisingly her favourite flowers were soft blackboards	Incongruent	Holland
42	They wanted somewhere bigger to live so they jumped at the chance when they heard that the building opposite them had recently advertised a cheap vacancy	Expected	apartment
42	The young couple wanted somewhere bigger to live so they were looking out for a cheap	Neutral	apartment

	vacancy		
42	They wanted somewhere bigger to live so they were looking out for notices advertising a three-bedroom semi-detached, cheap vacancy	Unexpected	apartment
42	They wanted somewhere bigger to live so they jumped at the chance when they heard that the building opposite them had a recently advertised, cheap racehorse	Incongruent	apartment
43	Made from natural materials derived from clay, she was pleased with her recent purchase as she placed the flowers into the nicely decorated vase	Expected	ceramic
43	Natural materials derived from clay, such as porcelain or pottery, are most commonly used to make a nicely decorated vase	Neutral	ceramic
43	All the pottery sold in our shop is made using natural materials derived from clay, including all plates, cutlery, and the nicely decorated vase	Unexpected	ceramic
43	Made from natural materials derived from clay, she was pleased with her recent purchase as she placed the flowers into the nicely decorated horoscope	Incongruent	ceramic
44	After the bullying episode, no one felt as alone and betrayed as the scared, targeted, and tired victim	Expected	sad
44	After the incident, there was no one who was as quite depressed and heartbroken as the tired victim	Neutral	sad
44	After the bank robbery, no one was as apologetic, mournful, and conscience-stricken as the convicted, tired victim	Unexpected	sad
44	After the bullying episode, no one felt as alone and betrayed as the scared, targeted, and tired	Incongruent	sad

	curtain		
45	To direct the orchestra, the conductor held a wooden stick that looked a bit like a magic wand	Expected	baton
45	While the orchestra was putting on an incredible performance of Ludwig van Beethoven’s 5th symphony, he used what looked like a wand	Neutral	baton
45	To direct the orchestra, the conductor first flicked through some pages and then read relevant notes off his music wand	Unexpected	baton
45	To direct the orchestra, the conductor held a wooden stick that looked a bit like a magic seashore	Incongruent	baton
46	The assailant used a dagger with a 6-inch blade and a very sharp edge as his favourite weapon	Expected	knife
46	When in his office, he used his dagger to open the envelopes he received but besides that he also used it as a favourite weapon	Neutral	knife
46	Before heading out onto the field, the gardener skillfully sharpened the very long metallic blade of his favourite weapon	Unexpected	knife
46	The assailant used a dagger with a 6-inch blade and a very sharp edge as his favourite soup	Incongruent	knife
47	In an attempt to straighten them, the orthodontist fitted her teeth with grey brackets and then inserted a metallic, thin wire	Expected	braces
47	During her last visit to the dentist, which she could still recall only too vividly, she was left with some thin wire	Neutral	braces
47	After the dentist had stopped drilling, he inserted the white, thin wire	Unexpected	braces
47	In an attempt to straighten them, the	Incongruent	braces

	orthodontist fitted her teeth with grey brackets and then inserted a metallic, thin itinerary		
48	One of several animals with 12 incisors, 4 canines and 26 molars is one of the most ferocious howling predators in the Northern hemisphere, the wild grey wolf	Expected	teeth
48	One of several four-legged, furry animals with 12 incisors, 4 canines and 26 molars is the grey wolf	Neutral	teeth
48	One of several animals with 12 incisors, 4 canines and 26 molars is man's best friend, the domestic grey wolf	Unexpected	teeth
48	One of several animals with 12 incisors, 4 canines and 26 molars is one of the most ferocious canines in the Northern hemisphere, the wild grey leek	Incongruent	teeth
49	Moving their way through dirt and soil, the nature reserve is home to several kinds of wriggly, small worm	Expected	earth
49	Often covered in dirt and soil, and found all over mainland Europe, the nature reserve is home to several kinds of small worm	Neutral	earth
49	Often found rolling in dirt and soil, the rescue farm is home to several small worm	Unexpected	earth
49	Digging their way through dirt and soil, the nature reserve is home to several kinds of wriggly, small jeans	Incongruent	earth
50	A good food to eat if you need to watch your weight is a sugar-free, low-fat dairy product such as quark or Greek yoghurt	Expected	diet
50	She was told by her dietitian that a good food to eat if you need to watch your weight is organic yoghurt	Neutral	diet

50	A good food to eat if you need to watch your weight is lean, grass-fed wagyu yoghurt	Unexpected	diet
50	A good food to eat if you need to watch your weight is a sugar-free, low-fat dairy product such as quark or Greek towels	Incongruent	diet
51	The cocktail was made by mixing lemon juice, sugar and egg white into pure Scottish Highland whisky	Expected	sour
51	The cocktail that the woman ordered was made by mixing lemon juice, sugar and egg white into whisky	Neutral	sour
51	The cocktail was made by mixing lemon juice, sugar and egg white with anise-flavoured, green whisky	Unexpected	sour
51	The cocktail was made by mixing lemon juice, sugar and egg white into pure Scottish Highland deliberation	Incongruent	sour
52	The physician who treated her held the highest academic honours and was employed at the university first as a lecturer and later as an esteemed professor	Expected	doctor
52	The calm and competent physician who treated her at the hospital was also employed as an esteemed professor	Neutral	doctor
52	The physician who treated her loved animals and had originally been practising as an esteemed professor	Unexpected	doctor
52	The physician who treated her held the highest academic honours and was employed at the university first as a lecturer and later as an esteemed apple	Incongruent	doctor
53	The city is provided with electricity from fossil fuel	Expected	power

53	This mid-sized city in the south east of England is provided with electricity from local fuel	Neutral	power
53	The city is provided with electricity from solar fuel	Unexpected	power
53	The city is provided with electricity from fossil tape	Incongruent	power
54	She wasn’t sure what the word meant so she decided to look it up in her Oxford English web dictionary	Expected	definition
54	She wasn’t sure what the word meant and didn't know whom to ask, so she decided to check a web dictionary	Neutral	definition
54	She wasn’t sure what the word meant, so in order to look it up she opened a new window in her web dictionary	Unexpected	definition
54	She wasn’t sure what the word meant so she decided to look it up in her Oxford English web tyre	Incongruent	definition
55	They had to put luminous stickers on to prevent birds from flying into the colourless, big window	Expected	transparent
55	They had to take some rather drastic measures to prevent animals from crashing into the big window	Neutral	transparent
55	They had to take some measures to prevent birds from nesting directly under the roof in the nicely structured, sheltering, big window	Unexpected	transparent
55	They had to put dark stickers on to prevent birds from flying into the colourless, big tea	Incongruent	transparent
56	As a housewarming present, they brought their friends a nice bottle of red wine	Expected	gift
56	As a housewarming present, they brought their friends a nice and expensive red wine	Neutral	gift

56	As a housewarming present, they brought their friends a nice bouquet of red wine	Unexpected	gift
56	As a housewarming present, they brought their friends a nice bottle of red birds	Incongruent	gift
57	Sitting there on the end of his fingertip, it was hard to believe that his cherished pet chameleon was a type of tiny lizard	Expected	small
57	Sitting there on the end of his fingertip, it was hard to believe that his cherished pet was a type of tiny lizard	Neutral	small
57	Sitting there on the end of his fingertip, it was hard to believe that his cherished, feathered pet was a type of tiny lizard	Unexpected	small
57	Sitting there on the end of his fingertip, it was hard to believe that his cherished pet chameleon was a type of tiny violin	Incongruent	small
58	She baked a cake for her daughter's fifteenth birthday party	Expected	celebration
58	She baked a delicious cake consisting of three layers for her sister's upcoming wedding party	Neutral	celebration
58	She baked a cake for her daughter's upcoming baby party	Unexpected	celebration
58	She baked a cake for her daughter's fifteenth birthday hill	Incongruent	celebration
59	She realised that she had gotten lost in the city, so she decided to look up the directions back to her hotel on her mobile phone	Expected	internet
59	She realised that she had gotten lost in the city, so she decided to look up the directions back to her hotel on a phone	Neutral	internet
59	She realised that she had gotten lost in the city, so in order to look up the directions back to her hotel, she unfolded her phone	Unexpected	internet

59	She realised that she had gotten lost in the city, so she decided to look up the directions back to her hotel on her mobile fish	Incongruent	internet
60	Because the temperature was below zero degrees throughout their 4-day hike, everyone was wearing several layers of thermal gear and had their heads covered with a woolly, warm hat	Expected	cold
60	Because the temperature was below zero degrees throughout their 4-day hike across the Northern parts of Sweden, everyone was wearing several layers of thermal gear and a warm hat	Neutral	cold
60	Because the temperature was below zero degrees throughout their 4-day hike, everyone was wearing several layers of thermal gear and a wind-resistant, waterproof, zip-up, warm hat	Unexpected	cold
60	Because the temperature was below zero degrees throughout their 4-day hike, everyone was wearing several layers of thermal gear and had their heads covered with a woolly, warm knife	Incongruent	cold
61	She was always by his side, and there was nothing in the world he cared more about than his precious Bernese Mountain dog	Expected	companion
61	She was always by his side and never let him down, and there was nothing in the world he cared more about than his dog	Neutral	companion
61	She was always by his side, and there was nothing in the world he cared more about than his precious Siamese dog	Unexpected	companion
61	She was always by his side, and there was nothing in the world he cared more about than	Incongruent	companion

	his precious Bernese Mountain stage		
62	Meditation and yoga are two effective ways of looking after your mental and emotional health	Expected	mindfulness
62	Yoga exercises, especially if they are combined with regular meditation practice, are thought to be a good way to improve your emotional health	Neutral	mindfulness
62	The simplest meditation practice consists of simply paying close attention to your emotional health	Unexpected	mindfulness
62	Meditation and yoga are two effective ways of looking after your mental and emotional economy	Incongruent	mindfulness
63	After falling over 2.5 metres down the side of the boulder, the climber was in intense pain	Expected	accident
63	After being forced to end his climbing tour much earlier than planned, all he could feel was intense pain	Neutral	accident
63	After miraculously surviving a 2.5 metre fall, the climber was immensely grateful and overcome with intense pain	Unexpected	accident
63	After falling over 2.5 metres down the side of the boulder, the climber was in intense rules	Incongruent	accident
64	The cameras had no trouble detecting the driver who was racing down the inner city road at 80 mph in his flashy car	Expected	speeding
64	The cameras had no trouble at all detecting the man who was racing down the tree-laned inner city road with his flashy car	Neutral	speeding
64	The cameras had no trouble detecting the rider wearing no helmet who was racing down the inner city road at 80 mph with his flashy car	Unexpected	speeding
64	The cameras had no trouble detecting the driver	Incongruent	speeding

	who was racing down the inner city road at 80 mph in his flashy food		
65	The artist produced stunning greyscale drawings using only a well-sharpened, simple pencil	Expected	images
65	The artist produced absolutely stunning drawings using only a simple pencil	Neutral	images
65	The artist produced stunning paintings using only the very best watercolours combined with a simple pencil	Unexpected	images
65	The artist produced stunning greyscale drawings using only a well-sharpened, simple vampire	Incongruent	images
66	To get better grip before attempting the most difficult route in the bouldering hall, the young athlete covered her hands with powdery, white chalk	Expected	climbing
66	Before attempting the most difficult route in the bouldering hall which only very few people had the strength to complete, the young athlete prepared herself by putting on some white chalk	Neutral	climbing
66	Before attempting the most difficult route in the bouldering hall, the young athlete protected her feet by putting on grippy, white chalk	Unexpected	climbing
66	To get better grip before attempting the most difficult route in the bouldering hall, the young athlete covered her hands with powdery, white guitars	Incongruent	climbing
67	After making them run through a complicated maze, the rodent researcher brought over the cage that normally housed the laboratory’s grey rats	Expected	science

67	After making them move around for a while, the animal researcher brought over the container that normally housed the laboratory’s grey rats	Neutral	science
67	After making them move around the snowy landscape, the antarctic animal researcher brought over the cage that normally housed the laboratory’s domesticated, black-and-white and grey rats	Unexpected	science
67	After making them run through a complicated maze, the rodent researcher brought over the cage that normally housed the laboratory’s grey bells	Incongruent	science
68	He never talked to anyone about his deepest feelings, but only wrote about them in his well-guarded diary	Expected	secret
68	He was a very private person and only revealed his deepest feelings to his well-guarded diary	Neutral	secret
68	He never talked to anyone about his deepest feelings, so they remained buried in his troubled, well-guarded diary	Unexpected	secret
68	He never talked to anyone about his deepest feelings, but only wrote about them in his well-guarded pond	Incongruent	secret
69	Rather than filling the pages with important points from the lecture, she was occupied by drawing little figures into her lined, used notebook	Expected	sketch
69	Rather than paying attention to the lecture, she was occupied by drawing little figures onto the used notebook	Neutral	sketch
69	Rather than paying attention to the lecture, she was occupied by drawing little figures onto the	Unexpected	sketch

	previously distributed used notebook		
69	Rather than filling the pages with important points from the lecture, she was occupied by drawing little figures into her lined, used chestnut	Incongruent	sketch
70	She felt completely parched after her running race, so she grabbed her bottle and rapidly gulped down several sips of fresh water	Expected	hydration
70	She felt completely exhausted after her running race, so the first thing she did was to get some fresh water	Neutral	hydration
70	She was sweating heavily after her running race, so the first thing she did was to seek out a tree under which she could get some fresh water	Unexpected	hydration
70	She felt completely parched after her running race, so she grabbed her bottle and rapidly gulped down several sips of fresh night	Incongruent	hydration
71	The massive bridge that is currently being renovated is very strong and made out of iron and sturdy steel	Expected	construction
71	The bridge spanning the city's river that is currently being renovated is made out of sturdy steel	Neutral	construction
71	The brown, medieval bridge that is currently being renovated is made entirely out of sturdy steel	Unexpected	construction
71	The massive bridge that is currently being renovated is very strong and made out of iron and sturdy soup	Incongruent	construction
72	She housed several rescue animals on her homestead, including cows and pigs as well as clucking, shy chickens	Expected	sanctuary

72	She housed several rescue animals on her homestead, including horses and shy chickens	Neutral	sanctuary
72	She housed several rescue equine animals on her homestead, including horses, mules, and shy chickens	Unexpected	sanctuary
72	She housed several rescue animals on her homestead, including cows and pigs as well as clucking, shy olives	Incongruent	sanctuary
73	He could tell that she was upset by the tone of her trembling voice	Expected	emotion
73	He could tell that his wife was upset by her trembling voice	Neutral	emotion
73	He could tell that she was upset by her shaking arms and trembling voice	Unexpected	emotion
73	He could tell that she was upset by the tone of her trembling calendar	Incongruent	emotion
74	The zoologist admired their short hops and swift jumps, and she was proud to have domesticated several long-eared, sweet rabbits	Expected	pet
74	The zoologist admired their nice fur and swift gait, and she was proud to have domesticated several sweet rabbits	Neutral	pet
74	The zoologist admired their swift and powerful jumps, and she was proud to have domesticated several Australian, sweet rabbits	Unexpected	pet
74	The zoologist admired their short hops and swift jumps, and she was proud to have domesticated several long-eared, sweet aprons	Incongruent	pet
75	They hadn’t seen each other in ages and had many interesting stories to discuss, so they decided to meet up for a nicely brewed, enjoyable coffee	Expected	chat
75	They hadn’t seen each other in ages and had	Neutral	chat

	many interesting stories and events to discuss, so they decided to have an enjoyable coffee		
75	They hadn't seen each other in ages and had many interesting stories to discuss, so they decided to meet up at a local pub for an enjoyable coffee	Unexpected	chat
75	They hadn't seen each other in ages and had many interesting stories to discuss, so they decided to meet up for a nicely brewed, enjoyable temple	Incongruent	chat
76	Before mounting her horse and entering the arena, the professional rider put on her helmet and protected her hands by wearing two leather gloves	Expected	equestrian
76	Before mounting her horse and entering the impressively decorated arena, the professional rider put on her helmet and her leather gloves	Neutral	equestrian
76	To protect herself from an injury before mounting her horse and entering the arena, the professional rider put on her leather gloves	Unexpected	equestrian
76	Before mounting her horse and entering the arena, the professional rider put on her helmet and protected her hands by wearing two leather suns	Incongruent	equestrian

Note. Each probe word listed in this table appears after the corresponding sentence in the exposure phase as part of the relatedness judgement task (see Methods for details). Detailed spreadsheets containing the stimuli for Experiments 1-3 and Experiment 4 separately as well as all behavioural data is available on the OSF (https://osf.io/a8mj4/?view_only=0fe94de6d5ce4000975ca304945a6b91).
